# Artificial Intelligence Algorithms for Early Detection and Risk Stratification of Type 2 Diabetes Mellitus, Multimodal Bioinformatic Phenotyping, and Its Complications: A Technical Review of Classical Machine Learning, Deep Learning, and Post-Hoc Feature Attribution

**DOI:** 10.3390/bioengineering13101132

**Published:** 2026-09-28

**Authors:** Kattia Orozco-Romero, Jonas Pariona-Torres, Jose Cornejo

**Affiliations:** Universidad Tecnológica del Perú, Lima 15046, Peru

**Keywords:** artificial intelligence, machine learning, early detection, diabetes prediction, deep learning, feature selection, predictive models, risk stratification, diabetic complications

## Abstract

Type 2 diabetes mellitus (T2DM) affects hundreds of millions of people worldwide, and nearly half of all cases remain undiagnosed. This technical review, conducted under conducted under the Preferred Reporting Items for Systematic Reviews and Meta-Analyses (PRISMA) guidelines and structured around the Population, Intervention, Comparison, Outcome and Context (PICOC) framework, identifies and synthesises the evidence on artificial intelligence (AI) algorithms for the early detection of T2DM and the risk stratification of its complications, evaluating their performance against conventional diagnostic methods and characterising their clinical implementation contexts. A systematic search in Scopus with a four-stage screening process, synthesised across five research dimensions, yielded 255 records, of which 47 studies met the inclusion criteria (2025–2026). Ensemble methods based on gradient boosting, particularly particularly extreme gradient boosting (XGBoost), emerged as the dominant primary modelling approach, while logistic regression was the most frequently reported algorithm, predominantly as a baseline comparator, with area under the receiver operating characteristic curve (AUC-ROC) values ranging from 0.63 to 0.97. AI models consistently outperformed glycated haemoglobin (HbA1c), whose reported sensitivity for prediabetes was only 49%, reaching accuracies of up to 96.7%, although that upper figure comes from a narrowly defined cohort with a high baseline prevalence and does not represent general screening performance. Multimodal approaches achieved the highest discrimination, combining wearables, continuous glucose monitoring, and genomics (AUC 0.96 internal, 0.90 external, for the separation of self-reported glycaemic states), and a real-world clinical deployment integrated into electronic health records improved glycaemic control outcomes among high-risk patients. An assessment of the eight anchor studies with thePrediction model Risk Of Bias ASsessment Tool updated for AI (PROBAST + AI) found high concern regarding quality in six of them, driven mainly by cross-sectional designs and self-reported outcomes. Overall, AI stands out as a viable and scalable complement to conventional screening, although gaps remain in multicentre prospective validation, reporting standardisation, and algorithmic equity across populations.

## 1. Introduction

Type 2 diabetes mellitus (T2DM) is today one of the greatest challenges facing global public health, and the figures leave no room for doubt. In 2021, according to the International Diabetes Federation (IDF), some 537 million adults were living with diabetes; if current trends hold, that number will reach 783 million by 2045 [1]. The World Health Organization, with a different methodology and age range, puts the number of adults with diabetes at 830 million in 2022 [2]. Between 90% and 95% of those cases correspond to T2DM. The disease originates in insulin resistance that advances alongside the deterioration of pancreatic β cells and that, without timely intervention, can lead to microvascular complications, affecting the kidney, retina and peripheral nervous system, and macrovascular complications, affecting the cardiovascular system [3,4]. To the clinical burden is added the economic one. Global diabetes-related expenditure exceeded USD 966 billion in 2021, a figure that on its own justifies the search for more efficient and accessible forms of early detection [1].

Much of the problem lies in the fact that T2DM advances silently. By the time diagnosis arrives, almost half of the affected adults do not even know they have it [5]. That silent interval can last for years in the form of prediabetes, impaired fasting glucose (IFG) or impaired glucose tolerance (IGT), and it is precisely the window in which a change in lifestyle or early pharmacotherapy can prevent or delay established disease [6]. Conventional screening relies on three reference tests: fasting plasma glucose (FPG), glycated haemoglobin (HbA1c) and the oral glucose tolerance test (OGTT). They are reliable, yes, but none of them is cheap or convenient: they require fasting, costs and laboratory infrastructure that complicate their large-scale use, above all, where resources are scarce [6].

Faced with those limitations, artificial intelligence (AI) and machine learning (ML) algorithms have emerged as a promising alternative for early detection of some medical diseases [7]. Their advantage is concrete: they capture complex, nonlinear patterns in large volumes of clinical, demographic and lifestyle data that classical statistical methods do not exploit as effectively [8,9]. The context also helps. The growing availability of electronic health records (EHRs), primary care registries and population surveys, together with non-invasive wearables that monitor vital signs [10], has provided the substrate needed to train and validate high-resolution predictive models. On that basis, techniques such as Random Forest, support vector machines (SVM), ensemble learning methods and deep neural networks have reported AUC-ROC, sensitivity and specificity values comparable to or better than those of conventional tests in multiple independent cohorts [11,12,13].

The recent literature shows a remarkable diversity of approaches. Within classical ML, ensemble methods (AdaBoost, XGBoost or Random Forest combinations) have demonstrated robustness in the face of imbalanced datasets, relying on resampling techniques such as SMOTE [14,15]. Deep learning (DL) went further and incorporated unstructured sources: whole-body magnetic resonance imaging [16], chest radiographs [17] or free-text clinical notes read with large language models [18]. In parallel, explainable AI (XAI) set out to attack a persistent obstacle, the opacity of the models [19], with techniques such as SHAP (SHapley Additive exPlanations) and LIME (Local Interpretable Model-Agnostic Explanations); this transparency is an indispensable condition for bringing them into clinical decision-support systems (CDSSs) [20,21,22].

That said, such a volume of publication does not translate into equally useful reviews. The existing ones tend to remain within a single technological corner (classical ML only, DL only or EHR only), to rely on ethnically homogeneous populations (with the PIMA Indian Diabetes dataset and its 768 instances at the forefront) [8,9], or to focus on management and treatment rather than on early detection and prediction [23,24]. No recent technical review integrates, in a structured and comparable manner, the full spectrum of algorithmic families (classical ML, DL, XAI and multimodal models), measuring their performance against conventional methods and cataloguing the clinical and technological contexts in which they were validated. That gap leaves clinicians and biomedical engineers without a reference synthesis to guide informed adoption.

Added to this diversity are underlying methodological problems that make it difficult to compare studies and to carry them into practice. Four of them recur again and again. The first is severe class imbalance in training which, left uncorrected, biases models towards the majority class [14,25]. The second is insufficient validation: many works stop at a single training-test split, with no external or prospective validation. The third is the lack of standardisation of metrics, which prevents objective comparison [26]. The fourth is the limited geographic and ethnic representativeness of the data, which compromises generalisation [27]. These shortcomings call for a critical synthesis that not only catalogues the state of the art, but also appraises the methodological soundness of the evidence base as a whole.

This technical review sets out to review, compare and synthesise the AI algorithms developed and validated for the early detection of T2DM and the risk stratification of its complications, measuring their performance with standardised metrics, identifying the clinical contexts of implementation and pointing out the methodological gaps that remain open. Unlike previous work, it covers in a single effort the entire spectrum of algorithmic families (from classical ML to DL, XAI and multimodal models with NLP) under a single reproducible framework based on PRISMA, with a systematic search in Scopus restricted to open access publications from 2025 to 2026. The findings point in three directions: ensemble methods and DL architectures trained on clinical or multimodal data reach AUC-ROC values of 0.85–0.97, whereas models restricted to survey-level predictors remain in the 0.63–0.82 range; the integration of multimodal data substantially improves predictive capacity; and interpretability through XAI is emerging as the critical enabling factor for clinical adoption. The contribution claimed here is the synthesis, not the review method: the procedure followed is the standard PRISMA-guided one, and no methodological novelty is asserted for it. What is new is the object and the scope of the synthesis—the 2025–2026 window read as a single corpus across classical ML, deep learning, explainable AI and multimodal models, with the type of comparator recorded for every study, so that the minority that evaluate a conventional diagnostic reference in their own cohort can be told apart from those that compare algorithms with each other.

The objective aligns explicitly with the PICOC question of Section 2.1: a population of individuals with T2DM, prediabetes or at risk; intervention by means of AI, ML and DL algorithms; comparison against conventional diagnostic methods; the outcome of early detection or prediction; and the context of clinical and technological implementation. The work is furthermore framed within the institutional research line of the Universidad Tecnológica del Perú, “ICT, electronic, robotic and telecommunications applications for competitiveness, health, education and citizen security”, and contributes to the development of the basic STEM competence of the graduate profile through the critical analysis and technical synthesis of AI systems applied to health.

This study is structured as a technical narrative review. It does not pool effect sizes, as a quantitative systematic review or meta-analysis would; it compares the architectures, data modalities and clinical deployment contexts of recent artificial intelligence solutions.

The remainder of the article is organised as follows. Section 2 details the search and selection methodology: the PICOC framework, the search equation and the eligibility criteria. Section 3 presents the results around the five research questions. Section 4 discusses the findings against the state of the art and points out the priority lines of future research. Section 5, finally, gathers the conclusions.

## 2. Methodology

This technical review was designed and executed following the guidelines of the Preferred Reporting Items for Systematic Reviews and Meta-Analyses (PRISMA) protocol [28], widely recognised as the reference standard for guaranteeing rigour, reproducibility and transparency in reviews of the scientific literature. The methodological protocol integrates three components articulated sequentially: (1) the definition of the conceptual framework by means of the PICOC model, (2) the iterative formulation of the search equation with quantitative validation in Scopus, and (3) the screening process guided by predefined inclusion and exclusion criteria, documented by means of the PRISMA flow diagram. Each stage is described below with the level of detail required for independent replication. The review protocol was not registered prospectively. It was deposited retrospectively in the Open Science Framework before resubmission, and is available at https://osf.io/2qy7f (accessed on 7 September 2026), where it is flagged as a retrospective registration.

### 2.1. Research Questions

To guide the synthesis of evidence, one main research question and five subsidiary questions derived from the analysis of the PICOC elements were formulated (Table 1). This approach ensures that the retrieval and analysis of the studies respond directly to the stated objectives of the review, minimising the incorporation of the tangential or irrelevant literature.

### 2.2. Search Strategy: PICOC Framework and Retrieval Equation

The search strategy follows the PICOC model: Population, Intervention, Comparison, Outcome and Context. Each of the five elements defines one dimension of the research question, and each dimension becomes one Boolean block in the query. PICOC was preferred over PICO because of the fifth element. Context matters here: an algorithm for early detection cannot be judged apart from the setting in which it runs and the data that feed it, whether that be an electronic health record, a wearable device, a primary care consultation or a population screening programme. Treating each element as a separate retrieval block keeps the conceptual framework and the Scopus query in the same shape.

The terms of each block were compiled from the vocabulary of the research questions and from the synonyms, acronyms and orthographic variants in common use in the clinical and computational literature. Within a block the terms are joined with OR and combine abbreviations (T2DM, NIDDM, HbA1c), spelling variants (glycated haemoglobin/haemoglobin) and truncation with an asterisk for morphological variants (neural network*, decision tree*, wearable*); multi-word terms are enclosed in quotation marks so that Scopus matches them as exact phrases rather than as loose combinations of words. The blocks are joined with AND, so a record is retrieved only when it satisfies the five dimensions simultaneously, a design that favours precision over exhaustiveness. Table 2 presents the operational definition of each element, the terms assigned to it and the research question that it serves; for reasons of space each list is abbreviated with the notation [+N terms], and the complete equation is reproduced verbatim in Table 3.

#### Final Search Equation (Version 3)

The search equation was subjected to an iterative process of three versions until an adequate balance between sensitivity and specificity was reached. Version 1 (three blocks: P, I and O) was discarded because of its excessive breadth. Version 2 (five blocks with 15 terms per block) returned 1259 records, considered unmanageable for a technical review. Version 3, adopted as definitive, reduced the set to 255 records by means of a controlled adjustment of the number of synonyms in each block (P = 15, I = 14, C = 11, O = 8, Co = 12 terms). The final equation, executed over the TITLE-ABS-KEY fields of Scopus, is presented in Table 3: Two limits of what this iteration establishes should be stated explicitly. First, the reduction from 1259 to 255 records measures the effect of trimming the number of synonyms within the same five-block structure; it does not measure the effect of the conjunction between the blocks. Second, Version 1 relaxed that conjunction directly, requiring only the Population, Intervention and Outcome blocks and dropping Comparison and Context, and it was rejected because the volume of records it returned was unmanageable for a review of this scope; that volume, however, was not recorded at the time. The restrictiveness of requiring all five blocks to co-occur in the title, abstract or keywords was therefore observed during the design of the equation but is not quantified in this article, and quantifying it would require re-executing the relaxed equations, which has not been done.

### 2.3. Data Source and Applied Filters

The search was executed exclusively in Scopus (Elsevier), selected for its comprehensive coverage of the biomedical literature, its native support for complex Boolean equations over the TITLE-ABS-KEY fields and its recognised relevance in systematic reviews in the areas of biomedical engineering and artificial intelligence applied to health. The search was run on 14 June 2026. The filters documented in Table 4 were applied to the 255 records identified by Equation v3.

After applying these filters, the set of records eligible for the screening phase was reduced to 85 records, which constitutes the input to the selection process described in Section 2.5.

The decision to rely on a single database was deliberate, and its cost was measured rather than assumed. Every record retained after the filters was checked against PubMed by DOI through the NCBI E-utilities interface in September 2026: 81 of the 85 retained records (95.3%) and 43 of the 47 finally included studies (91.5%) carry a PubMed identifier, so a parallel PubMed search would have added little to what Scopus retrieved. The reverse direction is more informative. The four included studies without a PubMed record, published in Informatics and Health, Discover Public Health, Shiraz E-Medical Journal and Diabetes Mellitus, would have been lost under a PubMed-only strategy; three of them appear in journals that PubMed does not index at all, and one is the most cited study of the entire corpus (23 citations in Scopus Table 9). No record retained after the filters was published in an IEEE venue. This comparison runs in one direction only: it establishes what PubMed would have added to the Scopus result, not what a search executed natively in PubMed or IEEE Xplore might have retrieved beyond the present equation. The restriction to a single source is accordingly reported among the limitations in Section 4.

### 2.4. Eligibility Criteria

The inclusion and exclusion criteria were defined a priori on the basis of the PICOC framework and of considerations of methodological feasibility. Their sequential application guarantees that the final corpus includes only studies that are relevant, report performance data and are accessible in full text. 

#### 2.4.1. Inclusion Criteria

Six inclusion criteria were predefined, structured according to the five elements of the PICOC framework (population, intervention, comparison, outcomes and context) plus one methodological consideration, as summarised in Table 5. Although IC6 admitted original articles, systematic reviews and conference papers, the retrieved set made the distinction immaterial: the 85 records retained after the filters comprised 79 original articles and six reviews, with no conference papers, and none of the six reviews entered the final corpus. All 47 included studies therefore carry the Scopus document type Article, although one of them is, by content, a scoping review indexed under that type.

#### 2.4.2. Exclusion Criteria

Of the 85 records that survived the filters described in Section 2.3, three could not be retrieved in full text: the publisher version was behind a paywall in all three cases, even though Scopus tagged them as open access. The tag in question was “Green Open Access”, which records that a self-archived copy exists somewhere rather than that the publisher hosts an open version, and no accessible full text was obtained for any of the three within the search window. They are counted as reports not retrieved (*n* = 3) in the selection flow (Section 2.5) Table 7, leaving the 82 records that entered full-text screening. Of the 82 records retrieved in full text, two exclusion criteria were applied sequentially. The first excluded studies focused on forms of diabetes other than type 2 diabetes mellitus or prediabetes (for example, type 1 or gestational diabetes), leaving out 28 articles and reducing the set to 54. The second excluded studies whose main focus was treatment, glycaemic management or already-established chronic complications (retinopathy, nephropathy, neuropathy, diabetic foot), without a genuine early detection or prediction component, which excluded a further seven of those 54. In this framework, “early detection” encompasses both primary screening for undiagnosed T2DM or prediabetes and secondary risk prediction, detection or stratification of microvascular and macrovascular complications (e.g., diabetic retinopathy, nephropathy, and neuropathy) in people who already have the disease; the boundary is the task performed, not the stage of disease. After applying both criteria, 47 articles remained included in the review. The criteria and the number of records excluded at each step are summarised in Table 6.

**Table 6:** Exclusion criteria applied sequentially to the records retrieved in full text.

Code	Exclusion Criterion	Records Excluded
EC1	Studies focused on forms of diabetes other than type 2 diabetes mellitus or prediabetes (e.g., type 1 or gestational diabetes).	28
EC2	Studies whose main focus is treatment, glycaemic management or the clinical description, staging or management of an already established chronic complication (retinopathy, nephropathy, neuropathy, diabetic foot), without a genuine early detection or prediction component. The criterion turns on the task, not on the disease stage of the population studied: studies whose object was to predict, detect or stratify risk were retained even when the target condition was a diabetic complication, which is why risk-prediction models for diabetic kidney disease, diabetic retinopathy and peripheral neuropathy appear among the included studies.	7
	Total records excluded	35

### 2.5. Study Selection Process and PRISMA Flow Diagram

The selection process was carried out in four successive stages (Identification, Application of filters, Screening and Eligibility), in accordance with the PRISMA 2020 standard [28]. Figure 1 presents the flow diagram with the volumes of records at each transition and the quantified reasons for exclusion. Screening of titles and abstracts and of full texts was carried out independently by two reviewers (K.O.-R. and J.P.-T.); disagreements were resolved by consensus and, when they persisted, by arbitration of the third author (J.C.). No automation tool was used.

Table 7 summarises in figures the selection flow represented in Figure 1, with the number of records retained and excluded at each of the four stages.

**Table 7:** Quantitative summary of the study selection flow (PRISMA).

Stage of the Process	*n*	Notes
Records identified (Equation v3, no filters)	255	PRISMA Identification stage. Database: Scopus.
After applying the filters of Section 2.3	85	Year 2025–2026, MEDI, Article/Review/Conference Paper, Open Access.
Not retrievable in full text	−3	Paywalled despite the OA filter; confirms the limitation of the filter in Scopus.
Available for full-text screening	82	Effective input to the screening phase.
After EC1 (diabetes type ≠ T2DM/prediabetes)	54	28 records excluded: type 1 DM, gestational DM, other types.
After EC2 (no early detection/prediction)	47	7 records excluded: management, established complications.
FINAL INCLUDED STUDIES	47	Final corpus for the narrative synthesis.

To characterise the corpus bibliometrically, its distribution by journal (Table 8) and by number of citations in Scopus (Table 9) is summarised below for the 47 included studies. Of the total, 66% (*n* = 31) were published in 2025 and 34% (*n* = 16) in 2026, which reflects the recent acceleration of scientific output in the area.

**Table 8:** Distribution of the 47 included studies by publication journal.

Journal	No. of Studies
Frontiers in Endocrinology	10
Diabetes, Metabolic Syndrome and Obesity	4
Diabetes Research and Clinical Practice	2
Journal of Clinical Medicine	2
Other 29 journals (1 study each)	29
Total	47

**Table 9:** The 10 included studies with the highest number of citations recorded in Scopus (accessed June 2026). All ten entries belong to the corpus of 47 included studies; the bracketed numbers refer to the bibliography.

Scopus Citations	Year	Study
23	2025	Predicting diabetes using supervised machine learning algorithms on E-health records [29]
11	2025	Multimodal AI correlates of glucose spikes in people with normal glucose regulation, prediabetes and type 2 diabetes [30]
11	2025	Learning from the machine: is diabetes in adults predicted by lifestyle variables? [31]
11	2026	Development and validation of a machine learning model to predict comorbid hypertension in type 2 diabetes [32]
11	2025	Development and validation of a machine learning model for prediction of type 2 diabetes [33]
11	2026	Development and Validation of a Machine Learning-Based Predictive Model for Peripheral Neuropathy Risk [34]
8	2025	A Machine Learning-Based Prediction Model for Diabetic Kidney Disease in Korean Patients [35]
7	2025	Predicting the onset of chronic kidney disease (CKD) for diabetic patients with aggregated longitudinal EMR data [36]
6	2025	Development of a 5-Year Risk Prediction Model for Transition From Prediabetes to Diabetes [37]
5	2025	Artificial intelligence identifies individuals with prediabetes using single-lead electrocardiograms [38]

As for the geographic distribution of the corpus, the 47 included studies are concentrated in a small number of countries, as summarised in Figure 2.

### 2.6. Data Extraction

Data extraction was carried out independently by two reviewers (K.O.-R. and J.P.-T.) using a structured electronic template. Discrepancies were resolved by consensus and, when they persisted, by arbitration of the third author (J.C.). The extraction form was designed so that each group of variables responded directly to an element of the PICOC framework and to its associated research question, guaranteeing traceability between the extracted data and the questions guiding the review (Table 10):

### 2.7. Assessment of Methodological Quality

This paper is a technical narrative review: it maps algorithmic implementations and does not pool diagnostic accuracy, as a quantitative systematic review would. A formal risk-of-bias instrument was therefore not applied to all 47 included studies. It was applied to the eight anchor studies on which the comparative synthesis of Section 4 rests. Each was assessed with the model-development part of PROBAST + AI [39]: 16 signalling questions in four domains (participants and data sources, predictors, outcome and analysis), which for development studies yield a judgement of concern regarding quality rather than of risk of bias, plus three applicability judgements. The assessment started from a structured extraction of the passages of each full text that bear on every signalling question, prepared with the assistance of a large language model, and from a provisional answer drafted from those passages. The authors then reviewed the answers and the domain judgements against the full texts and take responsibility for the assessment reported in Table A3; the answers to the 16 questions for each study are given in Supplementary Table S1. Two limits should be clear. The assessment was performed once, not in duplicate by independent assessors, so no inter-rater agreement can be reported; it does not extend to the remaining 39 studies. PRISMA 2020 was followed for the reporting of the identification, screening and selection of studies. Methodological rigour and sources of bias, including validation optimism and dataset partitioning, are appraised qualitatively across the whole corpus in Section 4.6 and Section 4.7.

### 2.8. Evidence Synthesis

Given that the included studies present heterogeneity in the algorithms evaluated, the data sources, the target populations and the metrics reported, a structured narrative synthesis was chosen as the main method of evidence integration. The synthesis is organised around the five research questions defined in Section 2.1 and reports: (i) the distribution of algorithmic families, (ii) the range of performance metrics by type of algorithm, (iii) the clinical contexts of implementation and (iv) the comparison against conventional baselines. This is a technical review: PRISMA 2020 is followed for the identification, screening and reporting of study selection, but no effect estimate is pooled. Consequently, no meta-analysis, no subgroup or meta-regression analysis, no sensitivity analysis of a pooled result and no certainty-of-evidence rating (GRADE or equivalent) were undertaken, since none of these procedures applies in the absence of a statistical synthesis. The completed PRISMA 2020 checklist provided as Supplementary Materials marks those items accordingly.

The comparative synthesis of Section 3 and Section 4 rests on eight anchor studies [30,31,34,40,41,42,43,44]. They were selected purposively before in-depth extraction, with one requirement: that each of the five research questions, each population category of Section 3.1 Table 12 (established T2DM at risk of complications, prediabetes or altered glucose states, and general-population adults), each data modality and setting in the corpus (hospital electronic records, national surveys, wearables and continuous glucose monitoring, an integrated health system, a decentralised digital trial and a real-world deployment) and the principal algorithm families of the corpus (logistic regression, Random Forest and gradient-boosting ensembles, together with survival and multimodal models) had at least one study extracted in-depth. The tables of Section 3 and the PROBAST + AI assessment of Section 2.7 refer to these eight. The other 39 studies enter the synthesis through the fields tabulated in Table A1 and Table A2 and through the corpus-level counts of Section 3 and Section 4.

### 2.9. Limitations of the Methodology

The following limitations inherent to the methodological decisions adopted are acknowledged, and should be taken into account when interpreting the results (Table 11):

**Table 11:** Methodological limitations identified and mitigation strategies.

Limitation	Description and Mitigation
Single-database bias	The restriction to Scopus may exclude studies indexed exclusively in PubMed, IEEE Xplore, Web of Science or Google Scholar. This decision was motivated by the need to control the volume of records and to guarantee the reproducibility of the Boolean equation.
Availability bias (open access)	The open access filter restricts the corpus to OA publications, potentially excluding high-impact studies in subscription journals. The restriction was retained to guarantee full-text availability for every screened record, and it is reported among the limitations in Section 4.
Restricted time window (2025–2026)	Limiting the search to recent publications maximises technological relevance but excludes foundational studies prior to 2025. The window was set deliberately to capture the current generation of AI models rather than the historical development of the field.
Subject-area bias (MEDI)	The restriction to the Medicine area may under-represent computational engineering or biomedical informatics studies with direct application to T2DM.
Methodological heterogeneity	The diversity in data sources, reported metrics and validation strategies limits direct comparability between studies and precludes a robust quantitative meta-analysis across all domains.

## 3. Results

In total, 47 studies met the criteria and entered this technical review after the PRISMA selection process, all from the 2025–2026 period. Output was concentrated above all in China (*n* = 22) and the United States (*n* = 8), with contributions from Canada, Spain and a variety of countries in Asia, Europe, Africa and Latin America. The subsections that follow organise the evidence by research question (RQ1–RQ5): target populations, algorithms used, performance against conventional methods, metrics reported and clinical contexts of implementation.

### 3.1. RQ1: Target Populations for AI-Based Early Detection and Risk Stratification

The reviewed studies applied AI tools across three broad population categories: people with already established T2DM at risk of complications, subjects with prediabetes or altered glucose states, and general-population adults captured in nationwide surveys. Table 12 summarises these categories and their main features.

**Table 12:** Classification of the target populations in the eight anchor studies.

Population Category	Main Characteristics	References
Older adults with established T2DM	Age ≥ 65 years; high burden of comorbidities (DPN, DR); sampling in tertiary metabolic centres	[34]
Large-scale national survey populations (USA)	NHANES 2007–2018; 29,509 non-pregnant adults; multiracial; lifestyle variables and anthropometric data	[31]
National survey populations in LMICs	NFHS-5 India 2019–2020; 1,087,006 adults ≥ 30 years; 52.96% women; urban and rural settings	[40]
Prediabetes/isolated impaired glucose tolerance (I-IGT)	1117 Chinese Han men aged 50–87 years; normal fasting glucose; I-IGT prevalence of 34.2%	[41]
Adults with T2DM complications (lower extremity risk)	48,209 adults newly diagnosed with diabetic polyneuropathy; Kaiser Permanente CA and CO, USA; mean age 64 years	[42]
Multi-ancestry prospective cohort (wearable + genomics)	NIH All of Us; 4589 adults ≥ 18 years; multi-ancestry; Fitbit step data + whole-genome PRS; follow-up for incident T2DM	[43]
Multimodal digital clinical trial (normoglycaemic + prediabetic + T2DM)	PROGRESS study; 347 deeply phenotyped individuals (174 NGT, 79 prediabetic, 94 T2DM); 48.1% under-represented (UBR) groups; 10-day CGM + 5 additional modalities	[30]
Real-world clinical cohort in specialist diabetes clinics	138,558 eligible patients with T2DM in 38 Italian diabetes clinics; AMD Annals network; no major complications at baseline	[44]

A critical epidemiological observation across the studies is the high prevalence of diabetes-related conditions in the populations studied. In India, “about 10.5% of adults aged 20–79 years are affected, corresponding to approx. 89,826,900 cases” [40], while in China the Han ethnic group exhibits the highest national diabetes burden. Specifically in older adult populations, “the burden of DPN is likely higher in older adults with T2DM, as advanced age was a well-established risk factor that can accelerate diabetes-related peripheral nerve degeneration” [34]. For prediabetes in particular, “recent epidemiological data indicate that the prevalence of prediabetes among Chinese men and women are 37.0% and 33.4%, respectively” [41].

The largest populations studied came from national health surveys: the NFHS-5 dataset from India covered 2,843,917 total respondents, of whom 1,087,006 adults aged ≥ 30 years were eligible for the analysis [40], and the analysis based on NHANES 2007–2018 included 29,509 participants representative of the adult population of the United States [31]. At the other end of the spectrum, the multimodal digital clinical trial PROGRESS included 347 deeply phenotyped individuals in the normoglycaemic, prediabetic and T2DM states [30], while the All of Us wearable cohort contributed 4589 multi-ancestry adults with prospective follow-up for incident T2DM [43]. Figure 3 shows the distribution of the included studies by population category and year of publication.

Notably, studies addressing populations in low- and middle-income countries (LMICs), particularly India and China, are increasingly represented in the literature, which reflects the disproportionate global burden of T2DM in these regions. A common limitation across the studies is the restriction of the populations to single-centre or single-country samples, which limits the generalisation of the results. For example, the NHANES-based study acknowledged that “the use of NHANES data is limited to the population of the USA, restricting the generalisability of our results” [31], while the Chinese prediabetes cohort was composed exclusively of Han men over 50 years of age, “which may affect the generalizability of our findings across different populations or ethnic backgrounds” [41].

### 3.2. RQ2: AI/ML/DL Algorithms Used for the Detection and Prediction of T2DM and Its Complications

The included corpus drew on a broad spectrum of algorithms: classical machine learning, ensemble methods, survival models and multimodal architectures based on deep learning. Logistic regression was the most frequent, almost always as a baseline, but it was the ensemble methods, above all XGBoost and Random Forest, that established themselves as the primary and best-performing model. Table 13 details the algorithm families identified.

**Table 13:** AI/ML/DL algorithms identified in the eight anchor studies.

Algorithm Family	Specific Methods	Methodological Characteristics	References
Classical ML	Logistic Regression (LR), Naïve Bayes (NB), Support Vector Machines (SVM), K-Nearest Neighbours (KNN)	Linear or kernel-based; interpretable; comparative reference for ensemble methods	[31,34,41]
Tree-Based Ensemble	Random Forest (RF), Decision Tree (DT), AdaBoost, Gradient-Boosting Machine (GBM)	Bagging and boosting; robust to non-linearities and missing data	[40,41,44]
Gradient Boosting	XGBoost, CatBoost, LightGBM	Sequential correction of trees; regularisation against overfitting; dominant in tabular clinical data	[30,31,34,40]
Ensemble Meta-learning	Super Learner (SL)	Cross-validation-based stacking of multiple base learners; loss-function-minimising combination	[42]
Survival/Time-to-Event ML	Penalised Cox PH (CoxNet), Random Survival Forest, survival SVM, HistGBSA, LightGBM-Cox, XGBoost-AFT, XGBSE variants	Adjustment for censoring; C-index as the main metric; suitable for predicting incident T2DM	[43]
Multimodal XGBoost + XAI (SHAP)	XGBoost + SHAP explainability; multi-feature configurations (CGM, microbiome, genomics, Fitbit, diet, EHRs)	Gradient boosting + Shapley explanations; leave-one-person-out cross-validation	[30,34,40]

XGBoost was the predominant primary model, with multiple studies converging on its superior performance for tabular clinical data. As described by [40]: “XGBoost is a gradient boosting framework excelling in speed and performance, developed by Chen and Guestrin. It operates by constructing an ensemble of decision trees sequentially, with each new tree correcting errors of previous trees, using advanced optimisation techniques and regularisation methods to reduce overfitting and improve model performance”. The selection of algorithms in most studies spanned “both traditional (LR, NB) and advanced ensemble methods (RF, XGBoost) to capture linear, probabilistic, and complex nonlinear relationships, enabling comprehensive comparison across modeling paradigms” [34].

Hyperparameter optimisation was systematically applied across the studies. The configurations included grid search with stratified k-fold cross-validation [31,34] and a three-framework comparison (random search, grid search and Bayesian optimisation) [40]. Notably, ref. [43] simultaneously evaluated twelve survival analysis models: “the models included penalized Cox regression with elastic net regularization (CoxNet), random survival forest (RSF), survival support vector machine (SVM), histogram-based gradient boosting survival analysis (HistGBSA), LightGBM with Cox loss, XGBoost with accelerated failure time (AFT) loss, XGBSE with Kaplan Neighbours, XGBSE with Kaplan Tree, extra survival trees, bagged survival trees, and a parametric Weibull AFT model.”

Shapley Additive Explanations (SHAP) were incorporated as the dominant explainability method. The Super Learner approach [42] represents the ensemble meta-learning paradigm: “a general loss-based ensemble learning method that uses cross-validation to combine multiple candidate risk predictors defined by ML algorithms (e.g., random forest) or parametric (e.g., logistic) models into a single risk predictor”. At the frontier of algorithmic complexity, ref. [30] deployed a multimodal XGBoost classifier “trained to separate normoglycemic individuals and individuals with T2D”, integrating variables of glucose spikes, food intake, microbiome features, genomic risk scores, Fitbit-derived activity and EHR conditions as distinct feature configurations. Figure 4 presents the frequency of use of each algorithm family.

The algorithmic landscape shows a clear progression: from classical statistical models towards ensemble and hybrid approaches, with SHAP-based explainability already a standard component of the most recent publications. It is a sign that the field is maturing. Predictive performance on its own is no longer enough: clinical implementability and regulatory acceptance today demand transparent model explanations.

### 3.3. RQ3: Comparison of AI Performance Against Conventional Diagnostic Methods

Where AI models are compared against conventional diagnostic tools, fasting plasma glucose (FPG), glycated haemoglobin (HbA1c) and clinical scoring systems, they generally show greater discriminative capacity and identify high-risk individuals whom the standard criteria misclassify. That comparison, however, is made far less often than the size of the corpus suggests, and the distinction matters: an algorithmic comparison and a clinical comparison answer different questions. Re-reading the comparator of all 47 included studies against their full texts shows that eight evaluate a conventional test, a published risk score or a clinical reference model in their own cohort and report its performance alongside that of the AI model; 10 further studies contrast AI with conventional practice only in prose, or against performance values published elsewhere; and the remaining 29 compare algorithms with one another or with a plain statistical baseline, with no clinical comparator at all. Table 14 therefore labels the type of comparison each entry rests on, and the statement above should be read as applying to the studies that measured a conventional reference in their own data.

**Table 14:** Comparison of the performance of AI models against conventional diagnostic or screening methods. The Population column states the cohort in which each result was obtained, because the reported figures are not comparable across cohorts of different composition and baseline prevalence. Each entry is labelled by the type of comparison it rests on: (a) the conventional reference was evaluated in the same cohort and its performance is reported alongside that of the AI model; (b) the conventional reference is invoked as a contrast, but its performance was not measured in this cohort and is taken from published sources or from its role as the diagnostic standard defining the outcome; (c) the comparator is another model used as an internal baseline. Two entries are of type (a), five of type (b) and two of type (c).

Type of Comparison	Population	AI Model Performance	Limitation of the Conventional Method	References
(b) Non-invasive AI (lifestyle/anthropometry) vs. laboratory tests	NHANES 2007–2018: 29,509 non-pregnant US adults, multiracial	XGBoost AUC 0.8168 using only lifestyle and anthropometry variables	“Current T2DM risk-screening methods often rely on laboratory-based tests, such as fasting glucose and HbA1c, which, while effective, can be resource-intensive, impractical for large-scale screening”	[31]
(b) AI vs. HbA1c for prediabetes detection	1117 Chinese Han men aged 50–87 with normal fasting glucose; I-IGT prevalence 34.2%	RF: accuracy 96.7%, AUC 0.96 (vs. the 49% sensitivity for HbA1c that [41] reports from the literature)	HbA1c: “mean sensitivity of 49% and a specificity of 79% in identifying prediabetes”	[41]
(b) AI vs. OGTT (gold standard)	1117 Chinese Han men aged 50–87 with normal fasting glucose; I-IGT prevalence 34.2%	ML models predict I-IGT without OGTT using routine EHR variables	OGTT is “technically complex, costly, and impractical for widespread routine screening”	[41]
(b) AI vs. clinical nerve conduction (DPN gold standard)	1450 elderly inpatients with T2DM, tertiary metabolic centre, Shanghai	XGBoost: AUC 0.951, F1 0.855 (test set); training AUC 0.973	“Nerve conduction studies represent the diagnostic gold standard; these methods are technically complex, costly”	[34]
(c) AI vs. previous ML model (external generalisation)	1450 elderly inpatients with T2DM, tertiary metabolic centre, Shanghai	XGBoost: training AUC 0.973, test AUC 0.951 (stable)	Previous model by Sun et al.: “AUC dropped substantially from 0.933 in internal validation to 0.811 in an independent test set”	[34]
(a) AI vs. clinical model (wearable + genomics augmentation)	4589 multi-ancestry adults (NIH All of Us) with Fitbit data and polygenic risk score	C-index: clinical 0.748 → +steps 0.774 → +PRS 0.867; sensitivity 75.7% → 85.7%	Clinical model alone (age, BMI, laboratory tests) C-index 0.748	[43]
(c) AI vs. standard logistic regression (Super Learner)	48,209 adults with diabetic polyneuropathy, two US health systems	SL AUC 0.845 (CI 0.826–0.863); simplified SL AUC 0.817; fewer alerts; PPV 70–80%	Naïve LR AUC 0.804 (CI 0.783–0.825): lower precision, more unnecessary assessments	[42]
(a) AI in EHRs vs. routine clinical care (outcome improvement)	138,558 eligible patients with T2DM, 38 Italian diabetes clinics	Patients with ML score: HbA1c ≤ 7.0% improved +9.0% vs. +4.5% in controls (*p* < 0.0001)	Standard care without an ML prediction tool	[44]
(b) Multimodal AI vs. HbA1c alone (stratification in prediabetes)	347 deeply phenotyped participants (of 1137 enrolled), PROGRESS, United States	Multimodal XGBoost: AUC 0.96 (internal)/0.90 (external); finer stratification within the same HbA1c range	HbA1c provides a point estimate; “substantial variability, potentially highlighting that our model is considering all available data”	[30]

The most convincing evidence of the superiority of AI over conventional methods comes from three angles. First, for the detection of prediabetes, conventional HbA1c testing shows a mean sensitivity of only 49% for identifying altered glucose states [41], whereas Random Forest achieved an accuracy of 96.7% (AUC 0.96) in predicting isolated IGT using routine clinical variables. That figure has to be read within its cohort: 1117 Chinese Han men aged 50–87 with normal fasting glucose and an I-IGT prevalence of 34.2%. Both the narrow demographic and the high baseline prevalence inflate the attainable accuracy, so the value cannot be transferred to general-population screening. Second, for the prediction of incident T2DM, a strictly clinical model (C-index 0.748) was progressively improved by wearable-derived physical activity data (C-index 0.774) and genomic risk scores (C-index 0.867), with sensitivity and specificity increasing “from 75.7% and 67.8% to 85.7% and 77.4%, respectively” when the PRS was added [43]. Third, the multimodal AI approach demonstrated that there is “substantial variability” in glucose spike profiles “for participants with the same value of HbA1c,” confirming that HbA1c on its own is insufficient for risk stratification within prediabetic populations [30].

Beyond predictive performance, ref. [31] underlined the practical advantage of AI-based screening: “none of the predictors requires blood sampling or imaging techniques and can be followed up with simple surveys,” making these tools viable for large-scale population screening in resource-limited settings. Likewise, ref. [44] demonstrated for the first time that “a validated diabetes complications prediction tool, developed using a machine learning approach and incorporated in electronic medical records, can improve quality of care”, a finding with direct implications for the transformation of clinical practice. Figure 5 contrasts the AUC of each anchor study’s AI model with the baseline that study reports; Table 14 labels the type of each baseline.

### 3.4. RQ4: Performance Metrics Reported for AI-Based Detection and Prediction

Performance reporting spanned a wide range of metrics, which reflect different evaluation philosophies: discrimination (AUC-ROC), balanced classification (accuracy, F1-score, precision, recall), clinical utility (positive predictive value, number of patients needed to evaluate, decision curve analysis) and calibration (Hosmer–Lemeshow test, Brier score, calibration curves). Table 15 summarises them.

**Table 15:** Summary of the performance metrics reported and selected results in the eight anchor studies.

Study	Best Algorithm	AUC-ROC	Accuracy	Sensitivity	Specificity	F1-Score	Additional Metrics
Peng et al. [34]	XGBoost	0.951 (test)	87.8%	83.4%	—	0.855	Brier = 0.087; H-L χ^2^ = 4.935, *p* = 0.668; DCA net benefit 0–0.9
Wang et al. [41]	LR (AUC)/RF (overall)	LR: 0.9731; RF: 0.96	RF: 96.7%	RF: 91.4%	—	RF: 95.7%	PPV = 99.1%, NPV = 95.6%; 8 algorithms compared
Riveros Perez & Avella-Molano [31]	XGBoost	0.8168	84.97%	11.62%	98.08%	—	5 algorithms; Youden threshold; CatBoost AUC = 0.5622 (worst)
Barman et al. [40]	RF Grid Search	0.6786	64.43% (RF Bayesian)	63.07%	—	40.93%	9 combinations (3 models × 3 optimisers); survey data limit the maximum AUC
Adams et al. [42]	Super Learner	0.845 (IC 0.826–0.863)	—	—	—	—	Simplified SL AUC = 0.817; naïve LR AUC = 0.804; PPV 70–80%; NNE < 2
Rahman et al. [43]	CoxNet (best C-index)	C-index 0.867 (+steps + PRS)	—	85.7%	77.4%	—	12 survival models evaluated; C-index range 0.677–0.867
Carletti et al. [30]	Multimodal XGBoost	0.96 (CI 0.94–0.98) internal; 0.90 (CI 0.82–0.97) external	—	—	—	—	AUPRC = 0.60 (CI 0.40–0.77) external; 6 glucose spike metrics (all *p* < 0.001)
Nicolucci et al. [44]	XGBoost	>0.80 all complications; 0.97 nephropathy	>70%	—	—	—	Clinical quality indicators as outcome proxies (ΔHbA1c, ΔLDL-C, ΔBMI)

AUC-ROC was the most universally reported metric, appearing in all eight selected studies. However, its interpretation must take the study design into account: the AUC values for models predicting complications in clinically enriched samples (0.85–0.97) are not directly comparable with the AUC values of models based on population survey data, where signal-to-noise ratios are inherently lower (0.63–0.68). As ref. [40] explicitly noted: “real-world survey data often suffer from limited biomarker availability and measurement variability, which can reduce the signal-to-noise ratio in predictive modelling. Unlike clinical datasets that capture detailed laboratory and physiological metrics in controlled settings, population-based surveys prioritise breadth over depth, which inherently limits maximum achievable predictive performance.”

Calibration metrics, which assess the agreement between predicted probabilities and observed outcomes, were reported in a minority of studies, but they represent an important complement to AUC. Ref. [34] demonstrated sound calibration both through the Hosmer–Lemeshow test (χ^2^ = 4.935, *p* = 0.668, indicating the absence of significant deviation) and through visual calibration curves closely aligned with the ideal reference line. The Brier score of 0.087 further confirmed excellent probability calibration for the XGBoost model. Decision curve analysis (DCA) was reported in [34,42], providing estimates of clinical utility across a range of decision thresholds, with the Super Learner approach yielding a positive predictive value of “70–80%” at alert thresholds of 30–50%, and “fewer than 2 patients would need to be evaluated to identify one likely to develop a lower extremity complication.”

Confidence intervals around the AUC estimates were reported in three studies [30,42,43], representing a methodological benchmark that most studies did not meet. The widening of the confidence interval from internal validation (AUC 0.94–0.98) to the external validation (AUC 0.82–0.97) observed in [30] illustrates the realistic performance attenuation to be expected when models are evaluated in genuinely independent cohorts. Figure 6 shows the distribution of AUC-ROC values by algorithm family.

The picture that emerges from the performance data is one of high discriminative potential when the models are trained on clinical or multimodal datasets (AUC 0.80–0.97), and a marked drop when they are restricted to survey data without laboratory biomarkers. Precision intervals (95% CI) around the AUCs continue to be reported inconsistently, which evidences the need for standardisation in future reviews.

### 3.5. RQ5: Clinical and Technological Implementation Contexts

Implementation was distributed across five main deployment contexts: hospital electronic medical records (EHRs/EMR), wearable device platforms, primary care, population screening programmes and multimodal digital clinical trials. The integration of AI into EHR systems was the most mature and clinically proximate context. Table 16 describes the clinical and technological contexts of implementation.

**Table 16:** Clinical and technological contexts of the AI tools in the eight anchor studies.

Implementation Context	Main Characteristics	References
Hospital EHRs/EMR (specialist care)	XGBoost integrated into the MetaClinic EMR; automatic alerts at every clinical encounter; 38 Italian diabetes clinics; AMD Annals network	[44]
Integrated health system EHRs (primary care)	Kaiser Permanente EMR (CA + CO); >5 million patients; common data model; longitudinal outpatient, pharmacy and laboratory data	[42]
Military hospital EMR (retrospective)	PLA General Hospital, Beijing; routine blood tests + anthropometry; outpatient setting; targeted screening to avoid the OGTT	[41]
National metabolic management centre EHRs	Standardised National MMC, Shanghai; structured follow-up system; lifestyle questionnaire + laboratory; older adults with T2DM; scalable to other MMC centres	[34]
Population-level health survey (digital screening)	NHANES (USA): digital questionnaire + physical examination; no laboratory requirement for the ML model; scalable to community settings	[31]
National survey, primary care in LMICs	NFHS-5 India; NPCDCS primary health centres; MUAC as a low-cost triage tool; integration into community outreach programmes	[40]
Wearable + genomics platform (mHealth/precision medicine)	NIH All of Us; intraday Fitbit step data via API; wearable Researcher Workbench; genomic PRS (338 DIAMANTE SNPs); adaptive mHealth interventions	[43]
Decentralised multimodal digital clinical trial	PROGRESS study; CGM (Dexcom G6) + Fitbit + food diary + gut microbiome + genomics + EHRs; fully remote/home-based; diverse cohort 48.1% UBR	[30]

Among the operationally most significant implementations was the Italian deployment reported by [44]: “the risk prediction tool was incorporated in the EMRs utilized by diabetes clinics (MetaClinic, Meteda SRL). At each encounter, the system automatically generated an alert to inform the clinician that the predicted probability of complication could be generated for the specific patient.” This represents a genuine clinical decision-support system (CDSS) operating in routine clinical care, not merely a research prototype.

At the wearable-genomics frontier, ref. [43] described a framework in which “minute-level activity records were transmitted through Fitbit’s Application Programming Interface (API), curated into structured tables, and integrated into the Researcher Workbench ‘wearable’ domain,” enabling the combination of passively collected real-life activity data with whole-genome sequencing for T2DM prediction. The authors propose this as “a practical framework for embedding genetic and physical activity data into clinical decision-support tools, tailoring mHealth interventions, and designing adaptive health programs that adjust activity targets in real time based on genetic predisposition and behavioral patterns.”

For resource-limited settings, ref. [40] identified a practical solution: “mid-upper arm circumference (MUAC) as a practical, low-cost anthropometric proxy strongly associated with central adiposity and insulin resistance. In resource-limited settings, MUAC could serve as an efficient triage tool to identify individuals for further diabetes screening, reducing reliance on weight-height measurement or BMI calculations.” This finding directly addresses the implementation barriers in primary care systems with limited digital infrastructure.

The most complex technological context was the decentralised digital clinical trial PROGRESS [30], which demonstrated “the feasibility of continuous multimodal data collection in a remotely monitored cohort, encompassing a range of glucose homeostatic conditions and with unparalleled representation of individuals historically underrepresented in biomedical research.” However, the simultaneous requirement of six data modalities (CGM, wearable, food diary, gut microbiome sampling, genomics, and EHRs) limits immediate clinical scalability.

A cross-cutting limitation across all implementation contexts is the predominance of retrospective designs and the scarcity of external validation (Section 4.6), which restricts the evidence on real-world scalability. As ref. [42] noted, “the use of this and similar prediction tools is limited by the availability of evidence-based practices and protocols to guide clinical decision making in response to risk information,” emphasising that algorithmic performance must be accompanied by integration into the clinical workflow in order to achieve meaningful patient impact.

## 4. Discussion

### 4.1. Algorithmic Landscape: Frequency Versus Performance

The results of this technical review confirm and extend a consensus that was already taking shape in the literature on AI and diabetes: gradient-boosting ensemble methods, and XGBoost in particular, are the dominant paradigm for prediction on tabular clinical data. Why? Because of several intrinsic advantages: they handle missing values natively, they incorporate L1/L2 regularisation that curbs overfitting in the modest samples typical of clinical cohorts, and they sit well with SHAP-based post hoc explainability, something increasingly relevant in regulatory frameworks such as the European Union AI Act.

Compared with the more traditional approaches, logistic regression and Random Forest, this consolidation around XGBoost in the 2025–2026 publications reflects a maturation of the field, driven by comparative performance and by how accessible the tooling ecosystem has become. One further finding deserves attention: survival ML (penalised Cox regression, random survival forests and XGBoost-AFT), infrequent in the included set, achieved the highest concordance indices (C-index up to 0.867) in predicting incident T2DM in prospective cohorts [43], a methodological gap that calls for dedicated research.

An important nuance distinguishes the frequency of use of each algorithm from its relative performance. Logistic regression is the algorithm that appears in the most studies (29 of 47; Figure 4), but almost always as a comparison baseline. When one examines which algorithm was the primary or best-performing model in each study (Figure 7), the picture changes: gradient-boosting methods, led by XGBoost, consolidate as the predominant family (13 studies), ahead of classical machine learning (11). Even so, the continued relevance of classical ML remains notable for outcomes with a strong clinical signal (nomograms for retinopathy, kidney disease or peripheral arterial disease), where regularised regression models achieved the best performance.

### 4.2. The Role of Data Richness in Performance

The observed AUC-ROC range (0.63–0.97) should not be read as a homogeneous distribution: each value makes sense within its methodological context. Studies trained on population surveys perform systematically lower (0.63–0.82). It is not that their algorithms are worse; it is that they have only anthropometric measurements, lifestyle and sociodemographic variables, without laboratory data, and that information predicts glycaemic status less well. Studies with rich clinical data, longitudinal EHR records or multimodal biological measurements achieve considerably greater discrimination (0.85–0.97); Figure 8 summarises this gradation between data richness and performance.

The value of 0.80 is used in this review as a descriptive reference band for reading the figures, not as a validated or normative threshold: no consensus AUC cut-off has been established for T2DM screening tools, and none is proposed here. Read that way, most studies with clinical or multimodal data sit above the band; the survey-based ones do so less consistently. The practical consequence is direct. Survey models work better as population triage, maximising sensitivity at the cost of precision, whereas clinical-data models make it possible to stratify risk individual by individual, with actionable positive predictive values of 70–80% [42].

The multimodal PROGRESS study [30] marks the performance ceiling identified by this review (AUC = 0.96 internal/0.90 external, for the separation of self-reported glycaemic states; Section 4.6), achieved by integrating six modalities: CGM, wearable activity, gut microbiome, genomics, dietary records and EHRs. But there is a practical nuance. The incremental analysis of the wearable-genomics survival study [43] shows that much of that gain is obtained with only two modalities, far easier to deploy: adding the step count from a wearable to a reference clinical model raised the C-index from 0.748 to 0.774, and adding a polygenic risk score took it to 0.867, a relative improvement of 15.9% over the clinical model, at a fraction of the complexity demanded by full multimodal integration.

The pattern points to a Pareto frontier between performance and scalability: the marginal gain from each additional modality falls rapidly after the first one or two non-invasive additions. For health systems constrained by infrastructure or budget, this supports betting on wearable-augmented clinical models as a pragmatic short-term objective, and reserving full multimodal integration for research settings or high-risk subspecialty environments, where collecting so much data is operationally feasible.

### 4.3. Coverage by Clinical Domain

Taken together, the included corpus is distributed across a broad spectrum of clinical outcomes (Figure 9): early detection and screening concentrate the largest share of the studies, followed by microvascular complications (kidney, retinopathy and neuropathy), cardiovascular and macrovascular disease, hospital readmission and other comorbidities.

Diabetic kidney disease was the most represented outcome in the extended corpus. Six studies addressed it, and their convergence is revealing: in a domain where the clinical signal is strong, regularised regression and ensemble methods reach a consensus that is uncommon in the rest of the literature. The work of [45] built a renal survival nomogram based on Cox regression in patients with biopsy-proven nephropathy, with a concordance index of 0.831. Ref. [36] took the same problem to aggregated longitudinal electronic health record data and obtained, with XGBoost, an AUC of up to 0.89 in its best subset.

A South Korean result nevertheless qualifies the dominant narrative: ref. [35] compared seven algorithms and found that the linear regularisation variants generalised better in external validation (AUC 0.827) than XGBoost, despite the latter’s slight internal advantage (AUC 0.810). There is therefore no universally superior algorithm; the best choice depends on the outcome. The three remaining studies [46,47,48], the last of these supported by natural-language processing over unstructured clinical text, agree on a common core of predictors, diabetes duration, glycaemic control and baseline renal function, with AUCs between 0.70 and 0.85.

Diabetic retinopathy contributed four studies that extend Table 13 with new algorithmic variants. Ref. [49] externally validated a LASSO and logistic-regression nomogram in older adults, with an external AUC of 0.788; the drop relative to its internal performance reproduces the same attenuation pattern already observed in [30]. Ref. [50] added the triglyceride–glucose index as a predictor and interpreted it with SHAP, reaching an AUC of up to 0.89 with XGBoost. The greatest discriminative power in the group corresponded to [51]: comparing eight algorithms for detecting vision-threatening retinopathy, XGBoost and LightGBM reached AUCs of 0.908 and 0.879, comfortably exceeding the 0.80 threshold. The fourth study breaks the mould. Ref. [52] did not predict risk but quantified changes in the microvascular parameters of the retina by means of AI-assisted segmentation of fundus photographs. Its inclusion is a reminder that AI applied to retinopathy is not exhausted by tabular models: direct image analysis is, in itself, a source of biomarkers.

Three studies reinforce the findings on diabetic neuropathy. Ref. [53] developed and multicentrically validated a model of painful peripheral neuropathy; in it, Random Forest offered the best overall discrimination (AUC 0.913, Brier 0.029), while logistic regression was the most sensitive (0.688), a reminder of the permanent trade-off between performance and interpretability. Ref. [54] took a different route: with the SUDOSCAN device, which measures electrochemical skin conductance, it assessed at once the risk of cardiac autonomic neuropathy and of nephropathy, and reported the highest AUC of the entire extended set, 0.97. That figure should nevertheless be read with caution, since it rests on a specific hardware technology and not on routine clinical variables. Finally, ref. [55] explored serum biomarkers, CCR2 and IDO1, as classifiers of peripheral neuropathy (AUC 0.728 to 0.786), evidence that subclinical inflammation offers a usable biological correlate beyond the purely algorithmic.

Hospital readmission appears as an application domain in its own right, with three studies. Ref. [56] trained a stacked ensemble on the public UCI Diabetes 130-US Hospitals dataset to predict 30-day readmissions and obtained an AUC of only 0.688. The figure is modest, and that modesty is informative: general readmission is a far more heterogeneous and noisy outcome than a well-delimited clinical complication. Ref. [57] narrowed the problem to patients with ischaemic stroke and concurrent diabetes and integrated markers of inflammation and metabolism, with which Random Forest and XGBoost reached an AUC of 0.78. The third case demands scepticism. Ref. [58] reported an AUC of up to 0.990 with convolutional neural networks in readmissions of older patients with heart failure and T2DM; an almost perfect value in a notoriously difficult problem suggests overfitting or data leakage, and obliges studies in this domain to meet a standard of external validation that is not met today.

Cardiovascular risk in T2DM was addressed by four studies that extend comorbidity beyond the microvascular. One datum challenges the idea that high performance requires the infrastructure of wealthy countries: [59], with data from the Ethiopian Public Health Institute and a Gradient-Boosting Machine, achieved an AUC of 0.96. Ref. [60] developed and externally validated a heart failure model whose AUC fell from 0.892 in training to 0.80 in external validation, another manifestation of the same decline that runs through this entire review. The two remaining studies drew on less usual data sources: ref. [61] applied natural-language processing to unstructured records to predict heart failure (AUC 0.69 to 0.74), and ref. [62] combined Medicare claims with electronic health records in older adults, with a C-statistic of 0.75, close to that of the baseline clinical model of [43] before incorporating wearable and genomic data.

Six studies substantially extend the evidence on screening and early detection described in RQ1, and reinforce the population diversity we already noted as a strength. On the transition from prediabetes to manifest diabetes, ref. [37] built a five-year risk model with seven algorithms; XGBoost and LightGBM reached an AUC of 0.865, well above the 0.67 of the simplest reference model. Ref. [63] stratified risk from the onset of prediabetes with XGBoost on electronic health records (AUC 81.59%, 95% CI 80.40 to 82.72) and explicitly recorded that its model outperformed stratification by HbA1c alone. Ref. [64] took a more pragmatic route: an index of only four predictors for women with previous pregnancies, with an AUC of 0.696, lower than that of the full model (0.760) but built solely from routine obstetric variables.

Not all the results were high. In a Chinese cohort of working-age adults, ref. [65] obtained a more modest performance (AUC 0.64 to 0.705), which again shows that broad population cohorts without specialised biomarkers run into a lower discrimination ceiling, just as we discussed for [40]. The group is closed by the scoping review of [66], which, by inventorying non-algorithmic tools (FINDRISC, CANRISK, and ADA-Risk Test, among others), confirms that these classical scales continue to coexist with machine learning models, above all in primary care, where the data infrastructure limits the more complex options. To this group is added a model trained on electronic psychiatric records [33], which, by means of XGBoost, identified patients with mental illness at risk of T2DM up to 2.7 years before their diagnosis (AUC-ROC 0.84), extending early detection to a population usually neglected in the literature.

Four studies illustrate how the input modalities for early detection are diversifying. The most striking is [38]: a single-lead electrocardiogram, processed with feature extraction and gradient boosting, is enough to identify prediabetes without a single laboratory test, which opens the door to mass screening with low-cost wearables. Ref. [67] combined a deep neural network with XGBoost in a hybrid explainable framework and reached an AUC of 0.94, among the highest of the population-based studies. The other two relied on conventional clinical data: ref. [29] compared KNN, Random Forest and logistic regression on electronic health records, and ref. [68] evaluated point-of-care HbA1c testing in a Chinese population, with the particularity of prioritising sensitivity and specificity over AUC. The set leaves a clear reading: early detection gains when heterogeneous sources converge, physiological, behavioural and clinical, and stops depending on the laboratory as the sole point of entry.

A final group of nine studies documents how AI approaches are extending towards comorbidities and less conventional data sources, always within the T2DM domain. In hepatology, ref. [69] modelled metabolic dysfunction-associated steatotic liver disease in an interpretable way, with an AUC-ROC of 0.873 using XGBoost. Ref. [70] built a nomogram for peripheral arterial disease in diabetic patients (AUC 0.826, C-index 0.795), and ref. [71] showed the prognostic value of complete blood count-derived inflammatory indices (SIRI, NLR, nMLR) for all-cause mortality in hospitalised patients, by means of Cox regression; both, together with [34,42] already discussed, extend the evidence on risk prediction in already established diabetes. Along the same lines, ref. [32] modelled concurrent hypertension in patients with T2DM using Random Forest (AUC 0.89 internal, 0.83 external), and ref. [72] transferred survival machine learning to the oncological field, predicting the survival of patients with uterine cancer and T2DM by means of a random survival forest (time-dependent AUC 0.823, C-index 0.90).

Other works bet on unusual data. Ref. [73] derived abdominal phenotypes from computed tomography, visceral fat and hepatic and pancreatic fat infiltration, to classify T2DM status with Random Forest and SHAP (AUC 0.72 to 0.74), a quantitative imaging modality rare in the rest of the sample. Ref. [74] analysed metabolomic risk associations in a large Finnish prospective cohort (AUC 0.68 to 0.74). Ref. [75], for its part, applied latent class growth mixture modelling to the HbA1c trajectories of the UK Biobank in order to isolate subgroups at high risk of complications, an approach that departs from the dominant binary classification.

The last case deserves a clarification: although its title speaks generically of “chronic diseases”, ref. [76] deals specifically with the early detection of T2DM with classical algorithms (Decision Tree, SVM, KNN, Naïve Bayes) on 768 records from an urban clinic in sub-Saharan Africa, and reaches an accuracy of 89% with an optimised neural network. Together with [59] in the cardiovascular field and [40] in the central synthesis, it reinforces a cross-cutting conclusion: high predictive performance is also attainable in low- and middle-income settings when the available data are of sufficient quality.

### 4.4. From Algorithmic Promise to Clinical Impact: The Translational Gap

The clinically most important finding of this review is not a metric but a quality-of-care outcome. The Italian multicentre deployment of an XGBoost-based CDSS integrated into the MetaClinic EMR system [44] showed that the proportion of patients reaching HbA1c ≤ 7.0% rose by 9.0 percentage points in the intervention group against 4.5 in the controls (*p* < 0.0001). It is the first rigorously controlled evidence that an ML-based complication prediction tool, embedded in the routine clinical workflow, produces measurable improvements in glycaemic control. That standard (algorithm inside the EHRs, automatic alerts at every encounter and prospective measurement of quality outcomes) is the benchmark of translational maturity to which the field should aspire.

Reality, however, is different: the great majority of the included studies did not go beyond retrospective development and internal validation (Figure 10), and left untouched the gap between the promise of the algorithm and its clinical impact. Closing it is not only a matter of technical performance; it demands research on usability, workflow redesign, accountability frameworks and institutional governance for AI-assisted decision support.

### 4.5. Limitations and Algorithmic Equity

One structural limitation recurs across the whole evidence base: development and validation are concentrated in homogeneous population samples, above all Chinese Han adults in tertiary metabolic centres [34,41] or adults in integrated health systems in the United States [42], which limits the extent to which the reported AUCs generalise to other ethnic, geographic and socioeconomic contexts. There are counterexamples worth highlighting: the explicit prioritisation of under-represented groups in PROGRESS (48.1% UBR) and the multi-ancestry composition of the NIH All of Us cohort [43]. But even there, none reported metrics disaggregated by ancestry subgroup, so possible performance disparities cannot be assessed.

For LMIC populations specifically, the NFHS-5-based analysis [40] showed that performance is attenuated but remains clinically useful with survey data (RF AUC 0.679), and identified mid-upper arm circumference (MUAC) as an inexpensive anthropometric proxy for community triage, with no need for infrastructure to measure BMI. Systematic attention to algorithmic equity (validation disaggregated by race, ethnicity, sex and socioeconomic status) should be a minimum reporting standard in future AI diabetes screening studies.

This review has several limitations. First, the heterogeneity across the 47 included studies (populations, algorithms, feature sets, and outcome definitions) precluded a formal meta-analysis; all the comparative synthesis is therefore qualitative. Second, that synthesis was anchored in eight representative studies, chosen before in-depth extraction to cover the five research questions, the population categories, the data modalities and settings, and the principal algorithm families (Section 2.8); being a purposive sample, it may not fully reflect the characteristics of the complete body of 47 articles. Third, publication bias probably inflates the observed performance, because works with low AUCs or failed models are indexed less frequently. A further limitation concerns the corpus itself, and it is stated here rather than left for a reader to find: two of the 47 included studies report no model performance metric at all. Reference [52] compares retinal microvascular parameters between patients with and without diabetic kidney disease, and ref. [75] models glycaemic trajectories and their associations; both report odds ratios or hazard ratios throughout, which measure association rather than the performance of a prediction model. Inclusion criterion IC4 asks for at least one quantitative performance metric, so on a strict reading these two sit at its edge. They were retained because their object is a detection or prediction task and they inform the clinical-domain coverage of Section 4.3, but they contribute no discrimination figure and are therefore absent from every performance aggregate in this review, including Figure 6 and Figure 8 and the ranges reported in Section 3.4. A further restriction of the search is acknowledged here without being quantified. The subject-area filter reported in Table 4 confined the search to records indexed under Medicine, which keeps the corpus clinically focused but excludes work that applies the same algorithms to T2DM and is indexed primarily under engineering or computer science. The number of records excluded on that ground was not measured, and no estimate of it is offered: establishing it would require re-executing the search without the filter. The direction of the resulting bias is nevertheless predictable, towards clinically framed evaluations and away from methods-driven ones, and it compounds the open access restriction discussed above rather than offsetting it.

Fourth, the PROBAST + AI assessment (Section 2.7, Table A3) covers the eight anchor studies only, was performed once rather than in duplicate, and was not extended to the remaining 39 studies, for which no instrument-based quality judgement is available. And fifth, the search was limited to a single database (Scopus) and to open access publications, so it may have left out relevant studies indexed in other sources, under restricted access or in languages other than English; something especially relevant if one considers that a large part of the global T2DM burden falls on countries where research is published mainly in other languages. The literature search was deliberately focused on Scopus-indexed, Open Access, Medicine-categorised articles from the 2025–2026 period. While an automated E-utilities check confirmed that 91.5% of included studies hold PubMed identifiers, restricting the search window to recent publications means that earlier foundational algorithms and non-Medicine indexed computer science proceedings were excluded. The choice favours a detailed picture of the current generation of deployable architectures, such as multimodal and NLP-based pipelines and SHAP-driven workflows, over the history of the field.

The restriction to open access deserves a more explicit treatment than a mention in the list above, because its effect is directional rather than random. Open access publication depends on the ability to pay an article processing charge or on institutional agreements that waive it, so the corpus is expected to over-represent research funded by public agencies and by institutions in high-income countries, and to under-represent work from settings without that support—precisely the low- and middle-income contexts where the T2DM burden is growing fastest and where a low-cost screening algorithm would matter most. The geographic concentration reported in Figure 2 is therefore not a neutral observation: it partly reflects where research is produced and partly reflects who can afford to publish it openly. The likely consequence for the conclusions is twofold. Reported performance is probably optimistic, since the same economic filter that selects for well-resourced institutions also selects for larger, better-curated datasets and for studies with positive results. And the coverage of implementation contexts is probably narrower than the true state of the field, which weakens any claim about generalisability to resource-constrained primary care. Neither effect can be quantified from the retrieved records alone; stating the direction of the bias is what the available evidence supports, and quantifying it would require a search without the access restriction.

### 4.6. Critical Appraisal of the Included Studies

The synthesis above reports what the included studies found; this subsection appraises how far those findings can be relied upon. The PROBAST + AI assessment of the eight anchor studies (Section 2.7) is summarised in Table A3 and discussed at the end of this subsection; what follows first appraises, for all 47 studies, the methodological features that were extracted from the full texts. Four of those features bear directly on how the reported performance should be read.

First, external validation is scarce. Re-reading the 47 full texts shows that 11 of them validate the model in a cohort independent of the one used for development, whether a different hospital, a separate registry or an independent prospective cohort [30,32,35,37,38,44,48,49,60,62,64]. The remainder rely on internal partitions: a train–test split, cross-validation or bootstrap resampling within a single dataset. Internal estimates are optimistic by construction, so the discrimination this corpus reports is, in aggregate, better than what these models should be expected to achieve on data they have not seen.

Second, the size of that optimism can be measured inside the corpus itself. Eight studies report a directly comparable pair of figures for the same model, one internal and one external [30,32,35,37,38,49,60,64]. The value falls in six of the eight and rises in two, with a median change of about −0.03 AUC; the largest drops are 0.851 to 0.785 [38], 0.96 to 0.90 [30] and 0.89 to 0.83 [32]. The penalty is modest but consistent, and it is observable only in the minority of studies that went looking for it.

Third, the partition behind a headline figure is not always declared. Several studies present a derivation or training-set value as the performance of the model, with the validation figure reported separately and lower: 0.725 against 0.698 [46] and 0.705 against 0.666 [65]. A review that aggregates headline values without checking which partition produced them obtains a range that is optimistic by construction, which is why the figures reported in Table 12 and Table 15 of this review were checked against the full texts rather than taken from abstracts.

Fourth, the design of the included studies is more varied than the field is usually credited with, and the variation runs along two axes that are worth separating. In time, the corpus is predominantly retrospective: 35 of the 47 studies describe themselves as retrospective, six as prospective cohorts, four as cross-sectional and one as a hybrid design that enrolled both ways, with the scoping review [66] having no primary design of its own. In provenance, however, the corpus is not predominantly single-centre: 17 studies draw on a single centre, department or clinic; 13 draw on two or more identified centres; and 15 draw on population-level sources—national surveys, biobanks, research cohorts, territorial registries, claims databases or public datasets—which belong to neither category. One study does not state its provenance [46]. Retrospective development and single-centre provenance therefore coincide in 16 of the 47 studies, roughly a third, and treating the two as a single characteristic would understate the geographic and institutional diversity of the evidence base while overstating how much of it comes from one clinical setting.

Read together with Section 3.3, where only eight of the 47 studies evaluate a conventional reference in their own cohort, these four features describe an evidence base that is growing quickly and reporting strong internal performance, but that has largely not been tested under the conditions in which it would be deployed. That is a statement about the maturity of the field, not a dismissal of it: the same studies also show which predictors and which data modalities carry the signal.

The PROBAST + AI assessment of the eight anchor studies (Table A3) adds detail to this reading. Concern regarding quality was high for six of the eight, low for one [42] and unclear for one [44], whose included article evaluates the clinical implementation of a previously developed model and reports its development only in summary. Three recurring features, rather than careless analysis, account for the high judgements. First, four of the eight [30,31,34,40] model a prevalent outcome with cross-sectional data, so that predictors and outcome are contemporaneous, although each is presented as risk prediction. Second, two use self-reported diabetes status as the label [30,31]; in [30] that label is the target of the multimodal classifier whose internal and external AUCs (0.96 and 0.90) are the highest in this review, so those figures measure the separation of self-declared groups rather than the early detection of biochemically confirmed disease. Third, four rely on complete-case analysis [31,40,41,43] and a fifth excluded incomplete records at enrolment [34]; one applies SMOTE without recalibration and before the data partition [40]; and none states explicitly whether predictors and outcomes were assessed blind to each other: two studies give no information on this [34,44], and in the other six the separation follows from laboratory or record-based measurement. Applicability concern was low for six and high for two [30,31], in both cases because the outcome assessed is a previously known diagnosis rather than incident or undiagnosed T2DM. The only anchor study with low concern in every domain [42] is a longitudinal cohort with a fixed prediction horizon, imputation with missing indicators, cross-validated ensemble selection confined to the training data and reported calibration: the features that the other seven, in different combinations, lack.

### 4.7. Sources of Heterogeneity and the Comparability of Reported Performance

The performance values collected in this review do not share a common scale, and the differences between them are only partly attributable to the algorithms. Four axes of heterogeneity account for most of the spread, and each moves the attainable ceiling independently of the model chosen.

Target population and prediction objective: The corpus combines studies that predict incident T2DM in a general or at-risk population with studies that predict, detect or stratify a diabetic complication in a population that already has the disease. The two tasks carry different base rates and different attainable ceilings, and a value obtained for one cannot be read against a value obtained for the other.

Cohort composition and baseline prevalence: Discrimination rises with the prevalence of the outcome and with the narrowness of the sample. The clearest instance in this corpus is the 96.7% accuracy obtained in 1117 Chinese Han men aged 50–87 with an I-IGT prevalence of 34.2% [41], already qualified in Section 3.3 and not transferable to general-population screening.

Predictor richness: Section 4.2 and Figure 8 document this axis empirically: models trained on survey-level variables, that is anthropometry, lifestyle and sociodemographics, sit in the 0.63–0.82 range, whereas models with laboratory panels, longitudinal EHR data or multimodal biological measurements reach 0.85–0.97. The gap tracks the information available to the model rather than the sophistication of the algorithm applied to it.

Validation strategy: As Section 4.6 sets out, only 11 of the 47 studies validate externally, and where an internal and an external estimate can be compared the external figure is usually the lower of the two. A comparison between an internally validated model and an externally validated one therefore favours the first for reasons that have nothing to do with either model.

The practical consequence is that the range reported in Section 3.4 describes what this literature reports; it is not a ranking. Two values within it are comparable only when the four axes above are comparable, which across a corpus of this heterogeneity is seldom the case. This is also the reason no pooled estimate is computed (Section 2.8), and the reason the comparison against conventional methods is labelled by type in Table 14 rather than presented as a single aggregate.

### 4.8. Explainability Beyond Post Hoc Attribution

The explainability reported across this corpus is almost entirely post hoc and attribution-based: SHAP appears in 22 of the 47 studies and LIME in one, both applied after training to rank how much each feature contributed. That is one family of methods among several, and the wider explainable AI literature has moved in directions this corpus does not yet reflect. Intrinsically interpretable designs embed the explanation in the model instead of reconstructing it afterwards, as DiaXplain does for T2DM diagnosis by pairing a deep model with a transparent reasoning path [77]. In continuous physiological monitoring the cost of producing the explanation becomes a design constraint in its own right, since an attribution computed too slowly is of no use at the point of care; evolutionary approaches to explainable classification of electroencephalographic signals illustrate that trade-off between fidelity and computational budget [78]. Furthermore, explanations are beginning to be produced as evidence-grounded natural-language reasoning rather than as feature rankings, with large language models constrained to answer from a defined evidence base [79]. None of these three works meets the eligibility filters of this review: they fall outside the Medicine subject area used in the search, and the latter two address mental-health monitoring rather than T2DM. They are cited here as the contextual literature and are not counted among the 47 included studies. Together they mark where explainability for clinical prediction is heading, from attributing a score towards justifying a decision.

## 5. Conclusions

The underlying conclusion of this review is that artificial intelligence algorithms already constitute a real and expanding advance for the early detection and risk stratification of T2DM, and they do so across highly diverse populations, clinical settings and technological contexts. Among the families evaluated, gradient-boosting ensembles and Random Forest are the most frequently reported as the best-performing model, with AUC-ROC values of 0.80 to 0.97 on clinical data and concordance indices of 0.74 to 0.87 in survival-based frameworks for predicting incident T2DM. That dominance is a finding about what this literature reports and about the preferences of its authors, not evidence that these algorithms are intrinsically superior. The studies differ in predictor availability, sample size, feature engineering, outcome definition and validation strategy, and those factors move the attainable performance independently of the model, as Section 4.6 and Section 4.7 set out. No causal attribution to the algorithm can be drawn from a narrative synthesis of studies that differ on all of them at once; establishing algorithmic superiority would require head-to-head evaluation on common data, which none of the included studies provides.

The ceiling was set by the multimodal approaches. By combining wearable physical activity, continuous glucose monitoring, genetic risk scores and gut microbiome profiling, they achieved the highest discrimination of the entire review (AUC 0.96 internal and 0.90 in independent external validation, for the separation of self-reported glycaemic states; Section 4.6). The message is clear: integrating heterogeneous data streams improves prediction far more than any isolated biomarker. In sum, AI can overcome the limits of the conventional criteria, from the low sensitivity of HbA1c for prediabetes, reported as 49% in the source cited by [41], to the logistical burden of the oral glucose tolerance test, and it positions ML-based screening as a feasible and scalable complement to standard clinical pathways.

Compared with previous reviews, almost always focused on the binary classification of T2DM in homogeneous Asian or North American cohorts, this work traverses five dimensions at once: populations, algorithms, comparative evidence, metrics and implementation contexts. And the difference is not only one of scope. A growing representation of studies from low- and middle-income countries (India) appears, alongside multi-ancestry prospective designs (NIH All of Us) and real-world clinical deployments on operational EHR systems. To this is added the consolidation of SHAP as the standard explainability framework: a sign that the field is maturing beyond pure performance, towards interpretable and regulation-compatible tools.

The practical implications are considerable. A model trained with routine clinical variables (with no additional laboratory tests, imaging or specialised equipment) is enough to flag the high-risk individuals who merit intervention in primary care or in the community. And the benefit is not theoretical: positive predictive values of 70% to 80% at realistic decision thresholds, together with the improvement in HbA1c control when the risk scores are integrated into the clinical workflow (9.0% versus 4.5% in the controls, *p* < 0.0001), show that these tools translate into measurable patient outcomes. In resource-limited settings, anthropometric proxies such as MUAC and national survey data open up avenues for population screening that do not demand laboratory infrastructure.

Several limitations qualify these conclusions and should guide future studies. The first concerns design and validation: 35 of the 47 studies describe themselves as retrospective and only 11 validate the model in an independent cohort, which systematically inflates the AUCs relative to what would be expected in a real prospective scenario. The corpus is not, however, predominantly single-centre: 17 studies draw on one centre, 13 on two or more, and 15 on population-level sources such as national surveys, biobanks, territorial registries or claims databases, as Section 4.6 details. The second is heterogeneity of reporting: the inconsistent inclusion of confidence intervals, calibration metrics or decision curve analysis precludes a formal meta-analysis and complicates any comparison between studies. The third is algorithmic equity, still little-examined; several authors acknowledged that their models might perform worse in marginalised or minority populations because of biases in diagnosis and data collection. The fourth concerns infrastructure: the computational and data demands of the most advanced multimodal approaches (CGM, genomics and microbiome) limit their scalability precisely where the diabetes burden is greatest.

Future research should prioritise the prospective, multicentre validation of the most promising algorithms in geographically and ethnically diverse populations, with standardised reporting: AUC with 95% confidence intervals, calibration, decision curve analysis and equity metrics stratified by race, ethnicity and socioeconomic status. The combination of continuous wearable monitoring with polygenic risk score points to the next frontier of precision prevention in T2DM, as suggested by the incremental improvements in C-index and sensitivity as those modalities were added to the reference clinical models. Even so, the most critical barrier is not algorithmic but translational: regulatory frameworks and clinical governance protocols will be needed to allow the safe and equitable deployment of CDSS tools integrated into EHR systems. This review identifies that as the true bottleneck between technical promise and population impact.

## Figures and Tables

**Figure 1:**
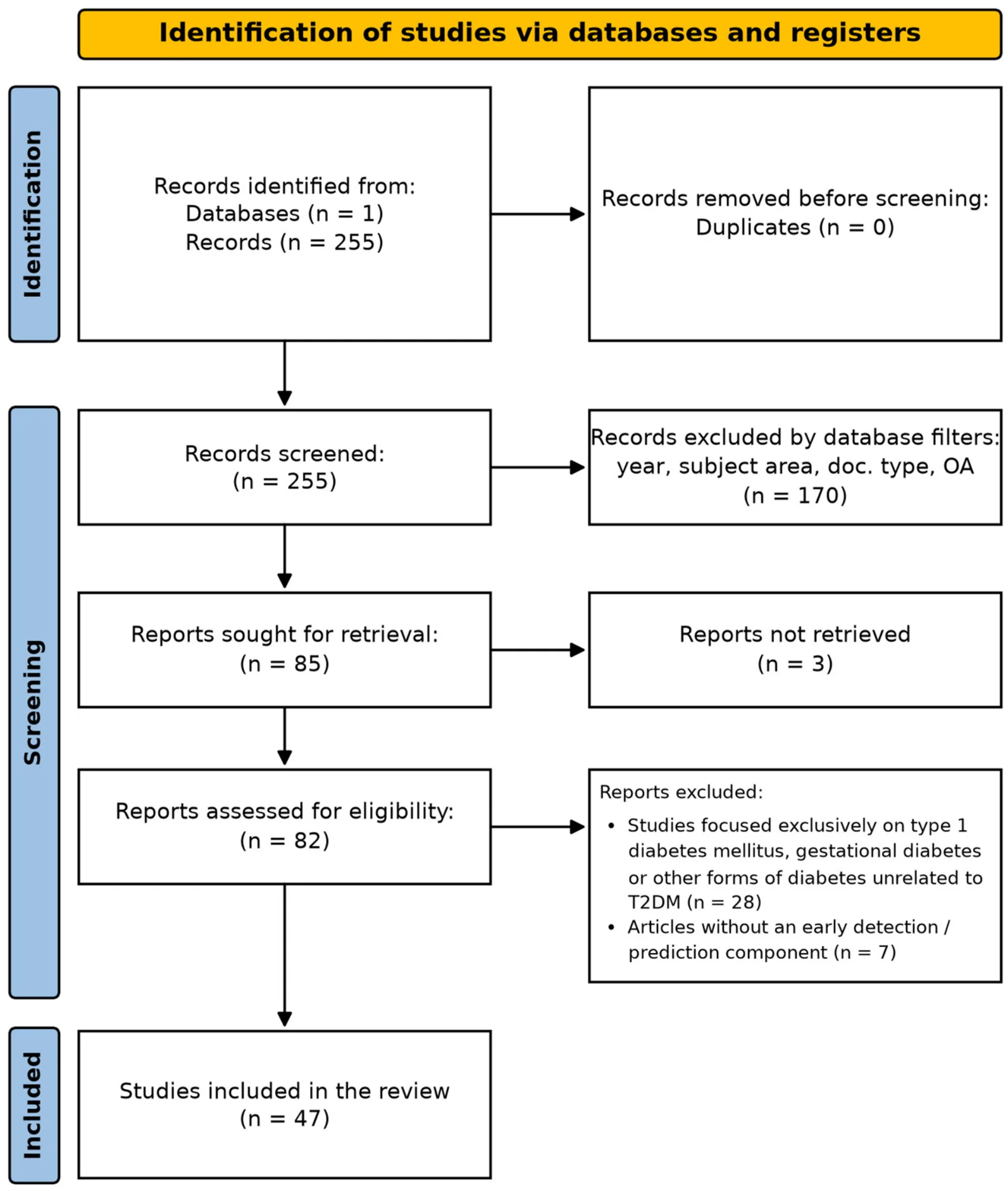
PRISMA flow diagram of the study selection process.

**Figure 2:**
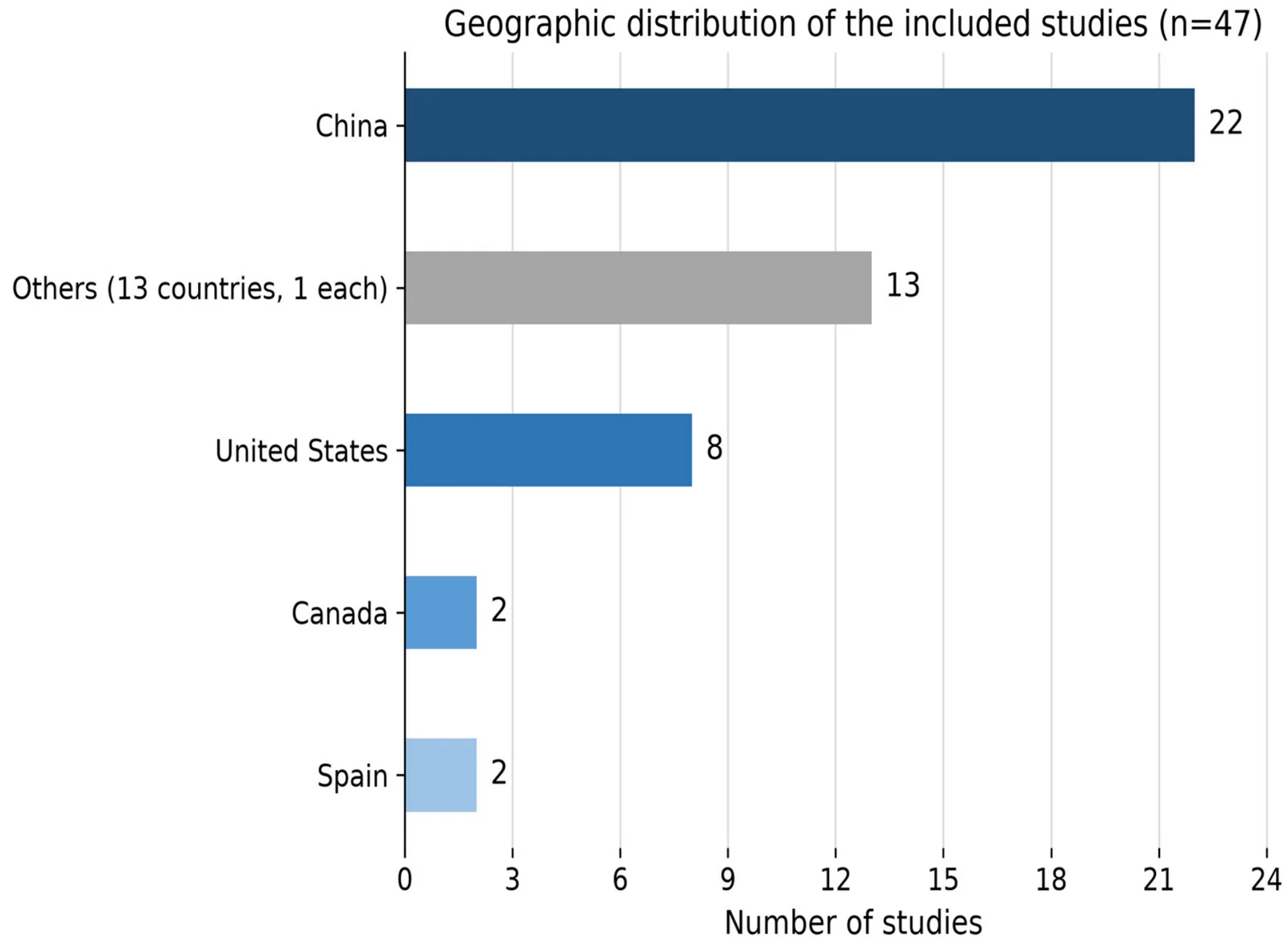
Geographic distribution of the 47 included studies. China accounts for 47% of the output (*n* = 22), followed by the United States (17%, *n* = 8).

**Figure 3:**
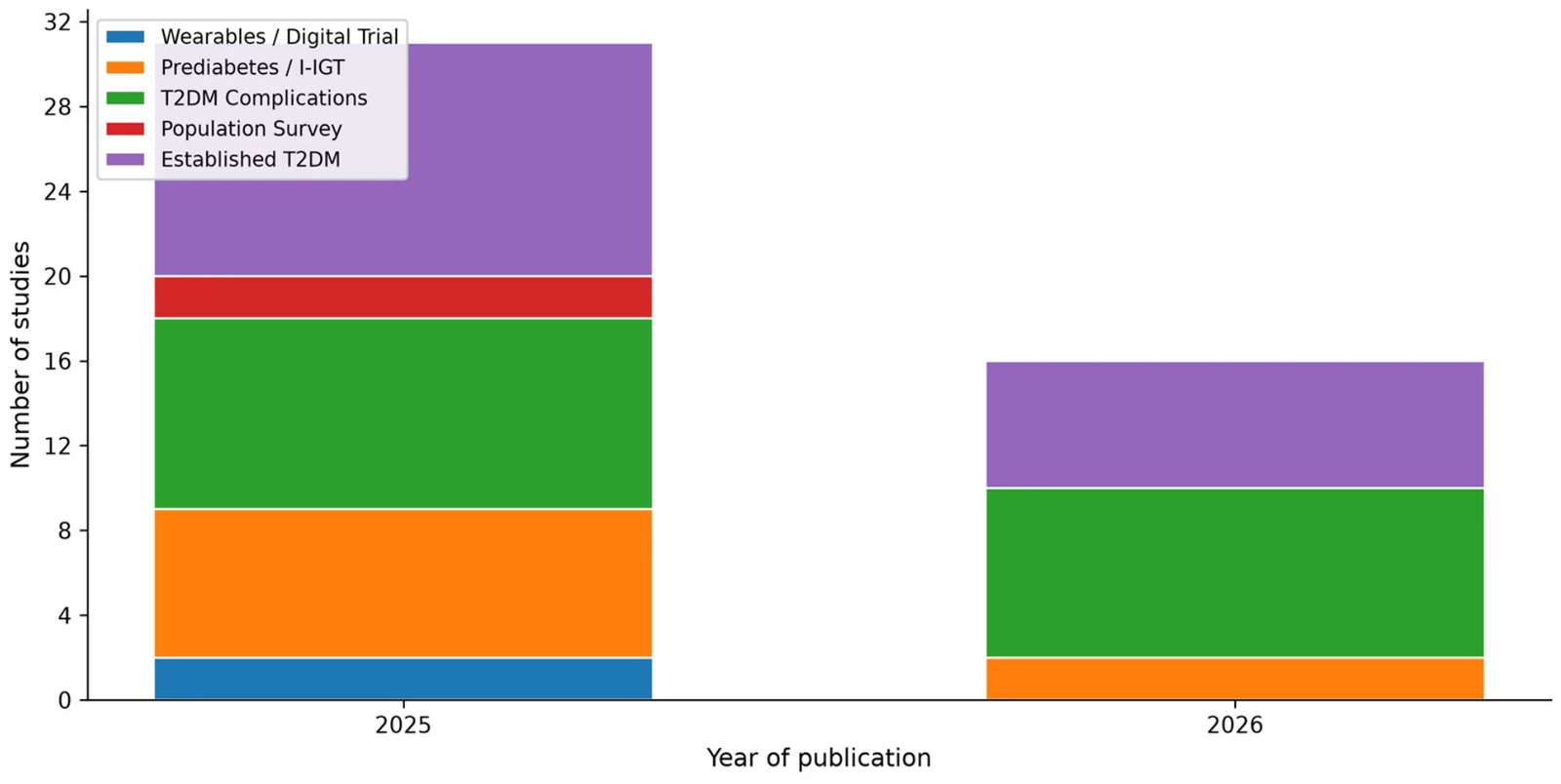
Distribution of the included studies by target population category and year of publication (2025–2026).

**Figure 4:**
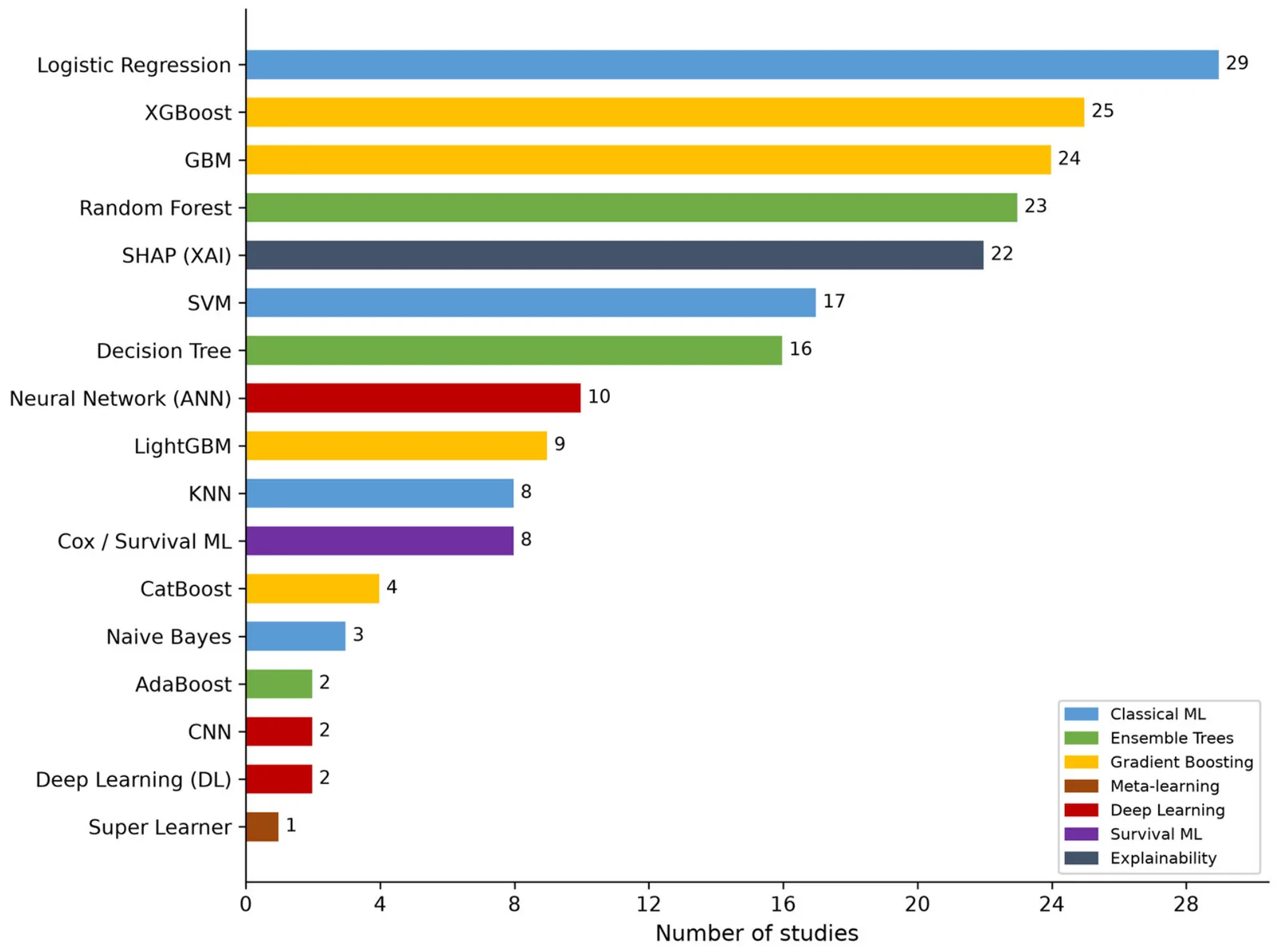
Frequency distribution of the AI/ML algorithms used in the included studies, grouped by algorithm family.

**Figure 5:**
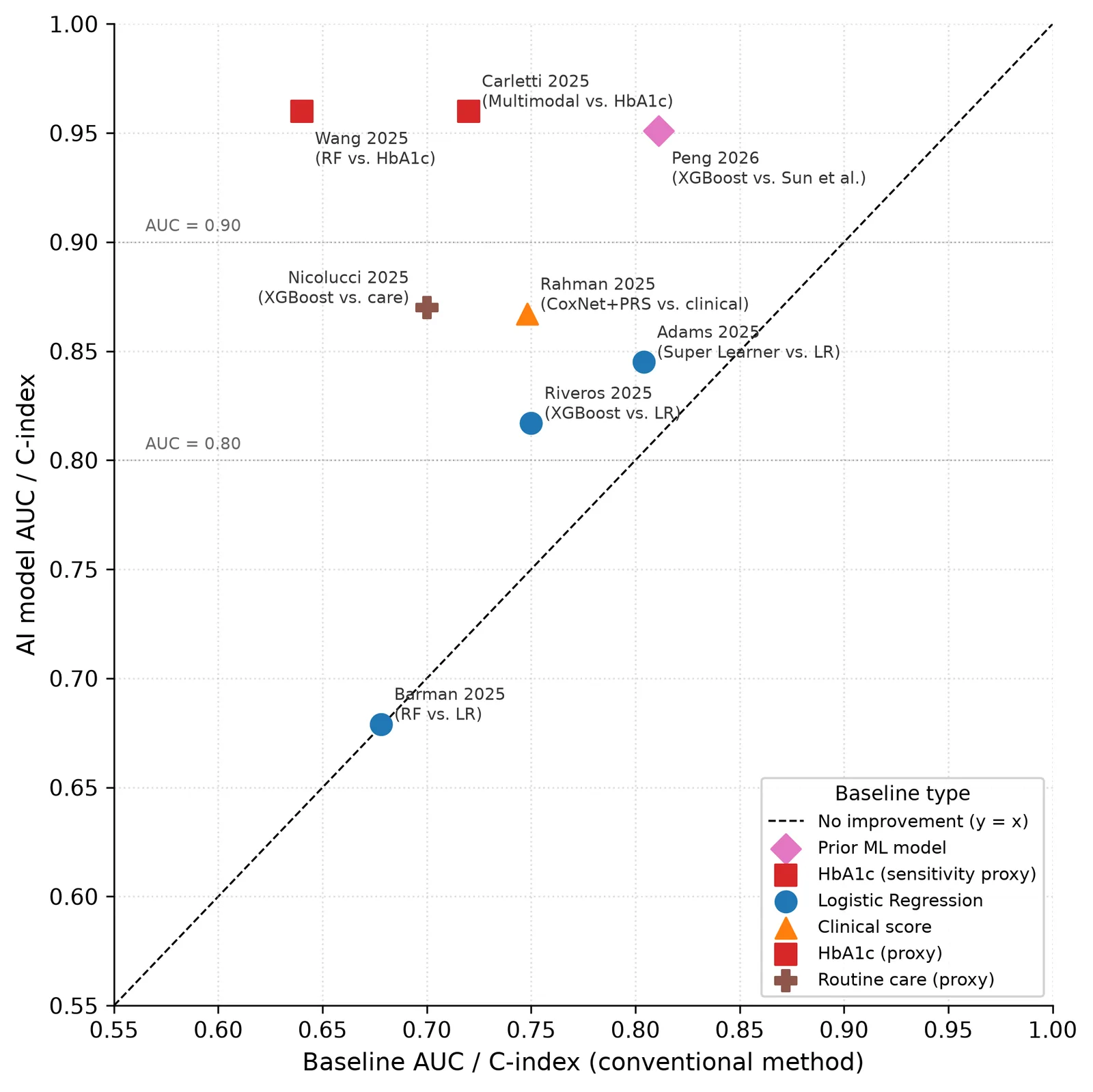
AUC of the AI model against the baseline reported in each of the eight anchor studies. The baseline is a conventional test or clinical score where the study evaluated one, and a logistic-regression or prior-model reference otherwise; the two HbA1c values and the routine-care value are proxies taken from published sources, not measured in the cohort (see the comparison types in Table 14). Points above the diagonal indicate that the AI model outperformed its baseline.

**Figure 6:**
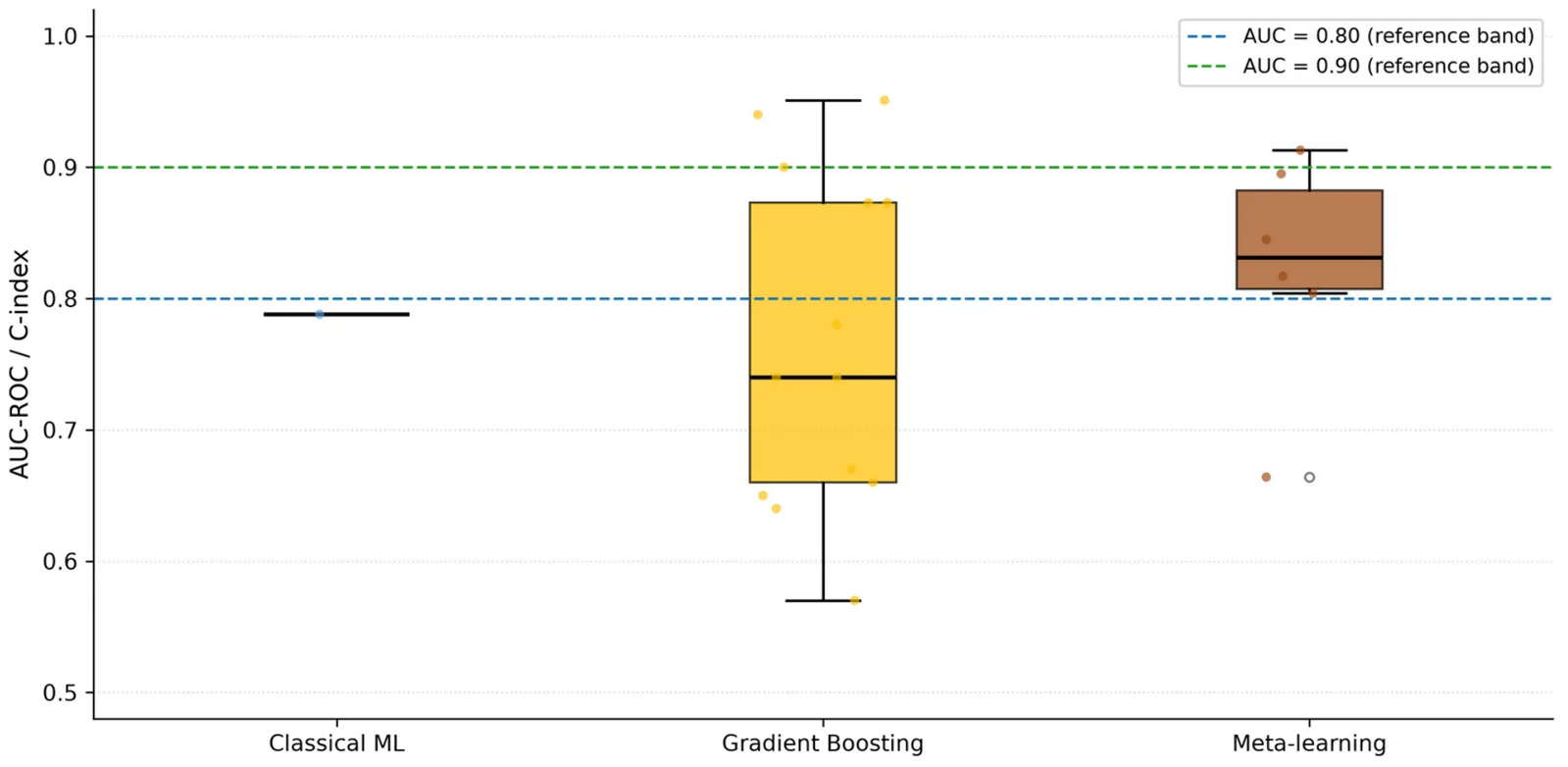
Distribution of the area under the receiver operating characteristic curve (AUC-ROC) values in the included studies, grouped by algorithm family. Each point is one AUC-ROC or C-index value reported in an included study (a study may contribute more than one value); point colours identify the algorithm family, and points are horizontally jittered to avoid overlap. Boxes show the median and interquartile range, and whiskers extend to 1.5 times the interquartile range; the hollow circle marks a value outside that range, which is also shown as a point. The dashed horizontal lines at AUC-ROC 0.80 and 0.90 are descriptive reference bands adopted in this review to aid reading; they are not validated diagnostic thresholds. The dotted lines are gridlines.

**Figure 7:**
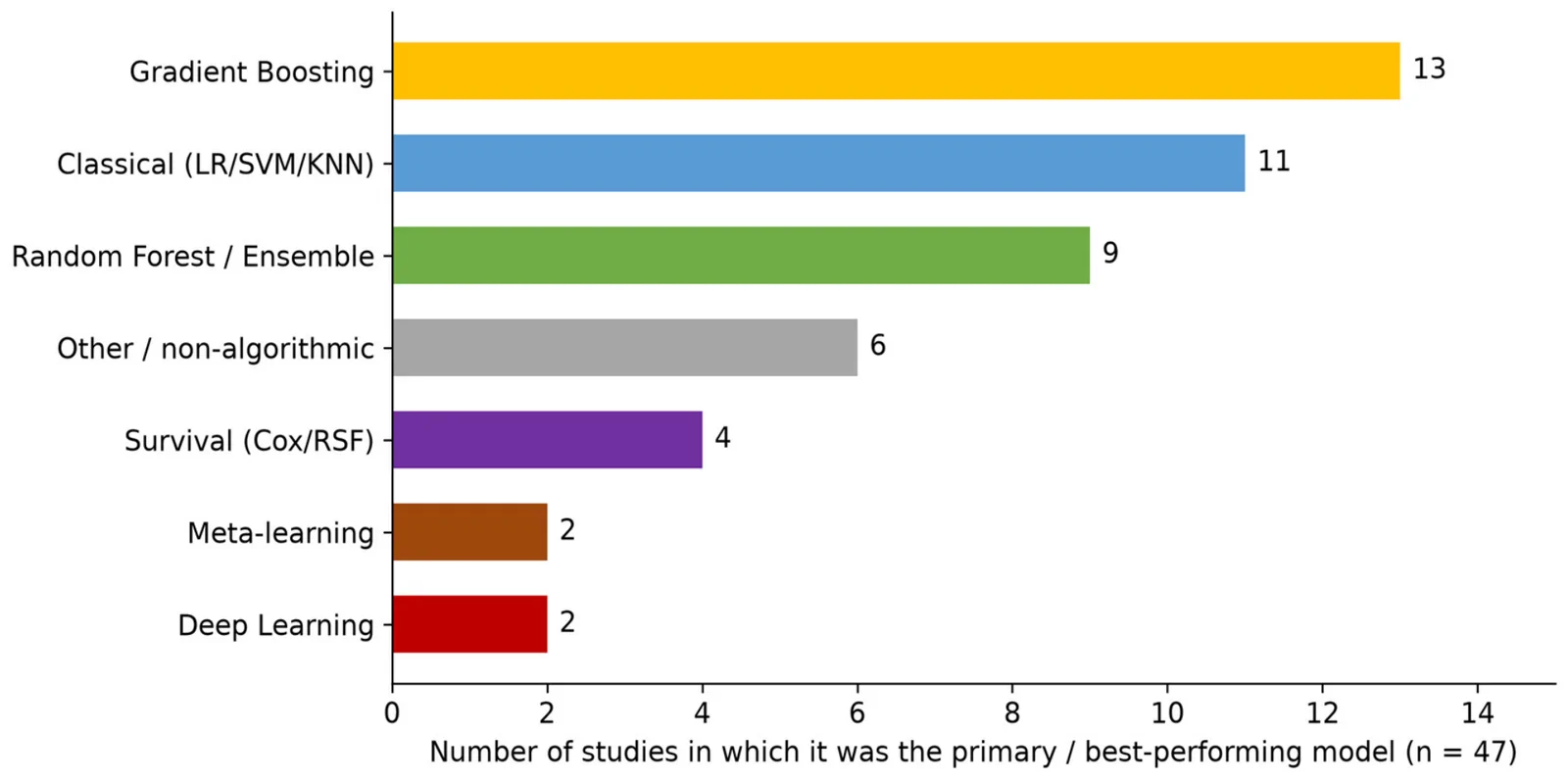
Primary or best-performing algorithm reported in each study, grouped by family (*n* = 47). Unlike Figure 4 (frequency of use), here a single family is counted per study: that of the best-performing model.

**Figure 8:**
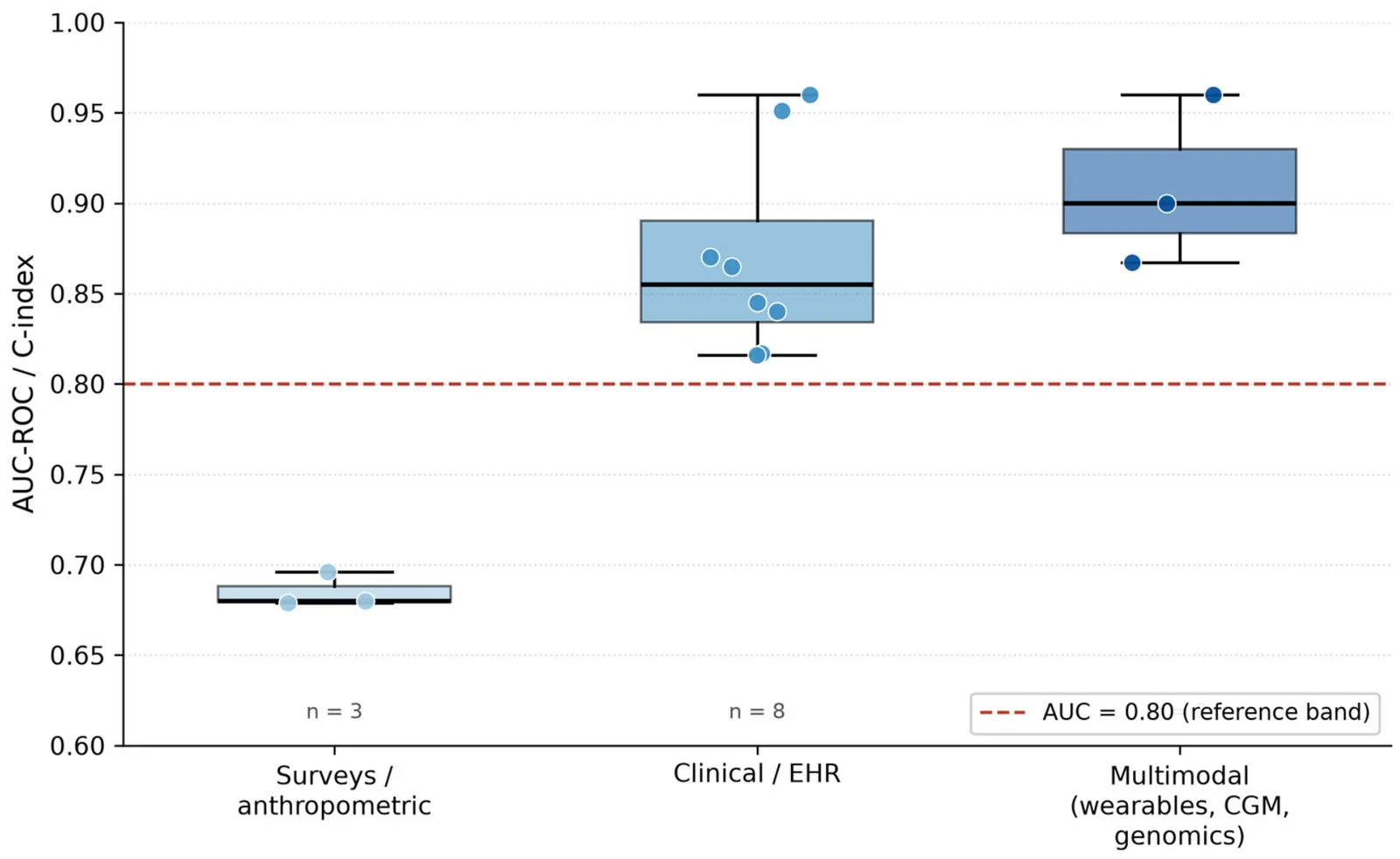
Distribution of the AUC-ROC/C-index values according to the richness of the data source (surveys/anthropometric, clinical/EHRs and multimodal) in the studies with reported performance. The dashed line at AUC 0.80 is the same descriptive reference band used in Figure 6, not a validated screening threshold; the values correspond to the figures reported by each study.

**Figure 9:**
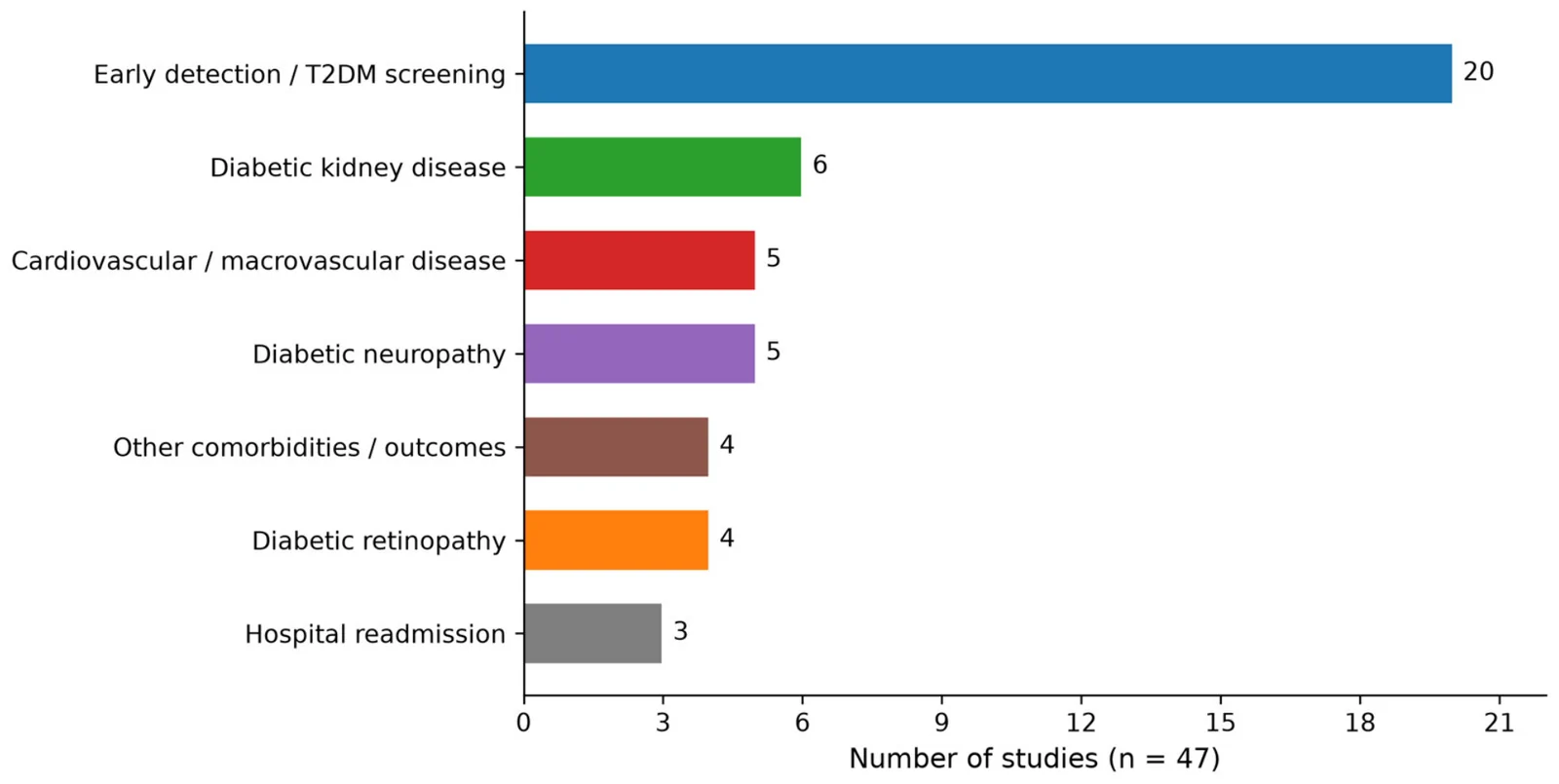
Distribution of the included studies by clinical domain/main outcome (*n* = 47). Note: each study is assigned to a single domain according to its main outcome; the counts reflect the total corpus (*n* = 47) and may differ from the thematic subsets discussed in Section 4.

**Figure 10:**
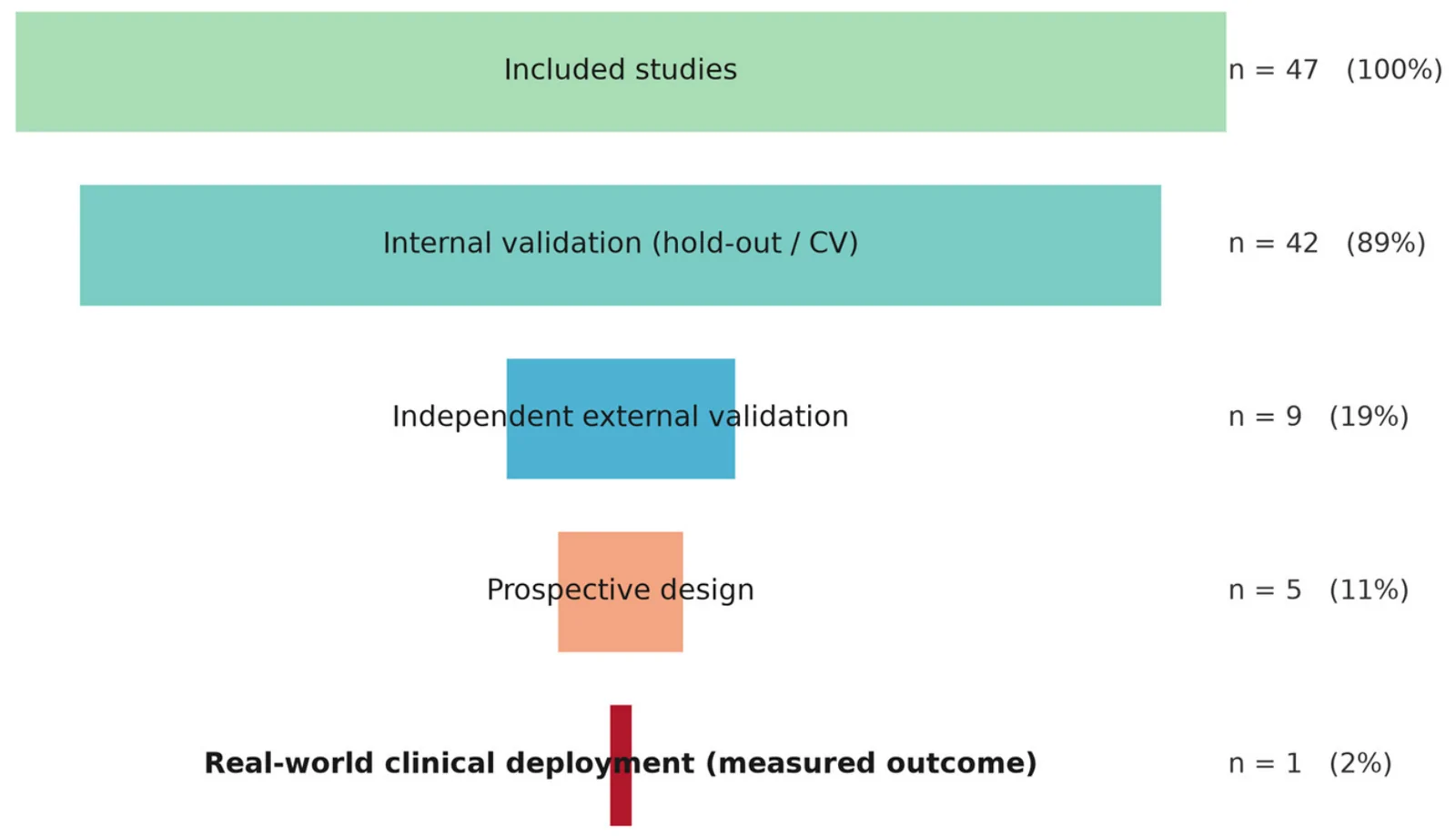
Number of studies (of the 47 included) reaching each translational maturity milestone, according to the classification of the extraction table (design and type of validation). The milestones reflect increasing levels of rigour and are not strictly nested subsets. A single study (MetaClinic [44]) achieved real-world clinical deployment with outcome measurement.

**Table 1:** Research questions formulated by means of the PICOC framework.

Code	Subsidiary Question	PICOC Link
Main	Which artificial intelligence algorithms have been developed and validated for the early detection of type 2 diabetes mellitus and the risk stratification of its complications, what is their performance compared with conventional diagnostic methods, and in which clinical contexts have they been applied?	P · I · C · O · Co
RQ1	In which populations (T2DM, prediabetes, at-risk subjects) has AI been applied for early detection?	P
RQ2	Which AI/ML/DL algorithms have been used for the early detection or prediction of T2DM and its complications?	I
RQ3	How does the performance of AI algorithms compare with conventional diagnostic methods or reference standards?	C
RQ4	Which performance metrics (sensitivity, specificity, AUC, accuracy) are reported for early detection/prediction by means of AI?	O
RQ5	In which clinical or technological contexts (EHRs, wearables, primary care, telemedicine, population screening) have these algorithms been implemented?	Co

**Table 2:** PICOC framework: operational definition and search terms by block.

Element	Operational Definition	Search Terms (OR)	Associated Research Question
P—Population	Patients with T2DM, prediabetes or at-risk subjects (impaired glucose tolerance, impaired fasting glucose)	“type 2 diabetes” OR “T2DM” OR “prediabetes” OR “impaired glucose tolerance” OR “impaired fasting glucose” OR “undiagnosed diabetes” OR “hyperglycemia” OR “diabetic patients” [+7 terms]	RQ1: In which populations has AI been applied?
I—Intervention	AI, ML and DL algorithms: neural networks, SVM, Random Forest, ensemble learning, gradient boosting, Decision Trees, data mining	“artificial intelligence” OR “machine learning” OR “deep learning” OR “neural network*” OR “convolutional neural network” OR “support vector machine” OR “random forest” OR “gradient boosting” OR “decision tree*” [+5 terms]	RQ2: Which algorithms have been used?
C—Comparison	Conventional diagnostic tests (FPG, OGTT, HbA1c) and classical statistical methods (logistic regression, reference standard)	“fasting plasma glucose” OR “oral glucose tolerance test” OR “glycated hemoglobin” OR “HbA1c” OR “logistic regression” OR “gold standard” OR “reference standard” [+4 terms]	RQ3: How does AI compare with conventional methods?
O—Outcome	Early detection, risk prediction, stratification, population screening, predictive analytics	“early detection” OR “early diagnosis” OR “risk prediction” OR “risk assessment” OR “risk stratification” OR “predictive analytics” OR “early screening” [+1 term]	RQ4: Which performance metrics are reported?
Co—Context	EHRs, wearables, mHealth, primary care, telemedicine, remote monitoring, decision-support systems, population screening	“electronic health records” OR “electronic medical records” OR “wearable*” OR “mobile health” OR “mHealth” OR “primary care” OR “telemedicine” OR “remote monitoring” OR “clinical decision support system” OR “population screening” [+2 terms]	RQ5: In which clinical contexts have they been implemented?

**Table 3:** Search strategy and final Boolean equation applied in Scopus.

PICOC	Search Block (Scopus, TITLE-ABS-KEY)
P	(“Type 2 Diabetes” OR “Type 2 Diabetes Mellitus” OR “T2DM” OR “Diabetes Mellitus Type 2” OR “Non-Insulin-Dependent Diabetes Mellitus” OR “NIDDM” OR “Prediabetes” OR “Pre-Diabetes” OR “Impaired Glucose Tolerance” OR “Impaired Fasting Glucose” OR “Diabetes Risk” OR “Undiagnosed Diabetes” OR “Glucose Intolerance” OR “Hyperglycemia” OR “Diabetic Patients”) AND
I	(“Artificial Intelligence” OR “Machine Learning” OR “deep learning” OR “Neural Network*” OR “Artificial Neural Network” OR “Convolutional Neural Network” OR “Support Vector Machine” OR “Random Forest” OR “Ensemble Learning” OR “Predictive Model*” OR “Data Mining” OR “Computational Intelligence” OR “Decision Tree*” OR “Gradient Boosting”) AND
C	(“Fasting Plasma Glucose” OR “Fasting Blood Glucose” OR “Oral Glucose Tolerance Test” OR “Glucose Tolerance Test” OR “Glycated Hemoglobin” OR “HbA1c” OR “Glycated Haemoglobin” OR “Clinical Diagnosis” OR “Logistic Regression” OR “Gold Standard” OR “Reference Standard”) AND
O	(“Early Detection” OR “Early Diagnosis” OR “Risk Prediction” OR “Early Identification” OR “Risk Assessment” OR “Risk Stratification” OR “Predictive Analytics” OR “Early Screening”) AND
Co	(“Electronic Health Records” OR “Electronic Medical Records” OR “Wearable*” OR “Mobile Health” OR “mHealth” OR “Non-Invasive” OR “Point of Care” OR “Primary Care” OR “Telemedicine” OR “Remote Monitoring” OR “Clinical Decision Support System” OR “Population Screening”)

**Table 4:** Filters applied to the records identified by the search equation.

Filter	Value Applied	Rationale and Associated Limitations
Publication year (PUBYEAR)	2025–2026	Captures the most recent developments in AI for T2DM detection. It implies that seminal studies prior to 2025 fall outside the inclusion corpus; they are documented in the discussion section as context.
Subject area (SUBJAREA)	Medicine (MEDI)	Ensures clinical focus. Potential limitation: studies primarily classified under Computer Science or Engineering that contribute algorithms with direct application to T2DM may be excluded. This is documented as a limitation of the search strategy.
Document type (DOCTYPE)	Article, Review, Conference Paper	Includes the formats with the greatest methodological density and reported evidence; excludes editorials, letters, errata and documents without performance data.
Open access (OA)	All Open Access	Guarantees full-text accessibility for the screening phase. It introduces an availability bias: high-impact studies published in subscription journals may not be represented. This is documented as a deliberate exclusion criterion and a possible limitation.

**Table 5:** Predefined inclusion criteria structured by PICOC element.

Code	PICOC Element	Criterion
IC1	P	Studies involving populations with confirmed T2DM, prediabetes, or subjects with risk factors (impaired glucose tolerance, impaired fasting glucose) as the target subjects of the detection or prediction intervention.
IC2	I	Studies addressing at least one computational or algorithmic approach to the early detection or prediction of T2DM—either by implementing, training or evaluating an AI, ML or DL model (including, but not limited to: artificial neural networks, CNN, SVM, Random Forest, gradient boosting, ensemble methods, Decision Trees or data mining models), or by systematically appraising the performance of such models alongside conventional risk-assessment tools.
IC3	C	Studies reporting an explicit or implicit comparison against a conventional diagnostic method (FPG, OGTT, HbA1c) or against a reference statistical model (logistic regression, clinical baseline).
IC4	O	Studies reporting at least one quantitative model performance metric: sensitivity, specificity, AUC-ROC, accuracy, F1-score, positive predictive value or equivalents.
IC5	Co	Studies conducted or validated in applicable clinical or technological contexts: electronic health records (EHRs/EMR), wearable devices, mHealth applications, primary care, telemedicine, remote monitoring or population screening.
IC6	Methodological	Original articles, systematic reviews with a primary computational component, and conference papers with reported performance results, published in English or Spanish between 2025 and 2026, in open access.

**Table 10:** Data extraction form structured by PICOC element and research question (RQ).

Form Category	Variables/Extraction Questions	PICOC Element → RQ
Bibliometric data	Authors, year, title, journal or conference, country of main affiliation, DOI, number of citations recorded in Scopus.	Corpus characterisation (cross-cutting to all RQs)
Target population	Type of population (established T2DM, prediabetes or at-risk subjects), source and size of the data, prevalence of T2DM in the sample, age range, country and ethnic composition.	P (Population) → RQ1
Algorithms evaluated	Algorithm family (classical ML, DL, ensemble), specific architecture, reported hyperparameters, validation strategy (hold-out, k-fold, external validation), handling of class imbalance (SMOTE, undersampling, weighting), explainability method (SHAP or other).	I (Intervention) → RQ2
Reference comparators	Conventional diagnostic or screening method used as a baseline (FPG, HbA1c, OGTT, clinical risk scores, standard logistic regression) and its reported performance against the AI model.	C (Comparison) → RQ3
Performance metrics	AUC-ROC, sensitivity, specificity, accuracy, F1-score, positive/negative predictive value, C-index, calibration metrics (Hosmer–Lemeshow, Brier) and 95% confidence intervals.	O (Outcome) → RQ4
Implementation context	Deployment setting (hospital EHRs/EMR, wearable platform, primary care, population screening, digital clinical trial), data source and considerations of clinical scalability.	Co (Context) → RQ5

## Data Availability

The original contributions presented in the study are included in the article. Further inquiries can be directed to the corresponding authors. The review protocol was registered retrospectively in the Open Science Framework, before resubmission and after screening had been completed, and is publicly available at https://osf.io/2qy7f (accessed on 7 September 2026). The registration is explicitly flagged as retrospective; the review was not prospectively registered, and PROSPERO does not accept reviews whose screening is already complete. The registration carries the title under which the manuscript was first submitted, “Artificial Intelligence Algorithms for Early Detection of Type 2 Diabetes Mellitus: A Technical Review of Classical Machine Learning, Deep Learning, and Explainable AI Approaches”; the title was broadened during peer review to reflect the scope of the included studies, and the search, eligibility criteria and corpus are unchanged.

## Supplementary Materials

The following supporting information can be downloaded at: https://www.mdpi.com/article/10.3390/bioengineering13101132/s1, Table S1: PROBAST + AI assessment of the eight anchor studies (answers to the 16 signalling questions with their supporting passages, and domain judgements); the completed PRISMA 2020 checklist. References [30,31,34,39,40,41,42,43,44] are cited in the Supplementary Materials.

## Appendix A

Table A1 lists all 47 included studies with the characteristics used in the synthesis. Country corresponds to the main affiliation, the same criterion used to build Figure 2; the algorithm column applies the same identification procedure used for Figure 4. Both are therefore consistent with those figures by construction. The n column reports the analytic sample as each study states it, verified against the full texts. Where a study reports a derivation and a validation cohort, both are given; where it reports an eligible population larger than the one analysed, the qualifier is stated; where no sample size is declared, the cell reads “Not stated”; and ref. [66], a scoping review of screening tools, has no primary sample of its own. The design column gives the temporal design (retrospective, prospective, cross-sectional or hybrid) and the provenance of the data (single-centre, multicentre or population-level source), verified against the full texts and counted in Section 4.6. Table A2 completes the characterisation with the four fields needed to follow the synthesis of Section 3.3 and Section 3.4 study-by-study: the data source, the type of comparator, whether validation was internal or external, and the headline performance metric with the partition it comes from. Table A3 reports the PROBAST + AI assessment of the eight anchor studies described in Section 2.7.

**Table A1:** Characteristics of the 47 included studies: country of main affiliation, analytic sample size, study design and AI/ML algorithms evaluated.

Ref.	Study	Country	*n*	Study Design	AI/ML Algorithms Evaluated
[34]	Peng J. (2026)	China	1450	Retrospective, single-centre	XGBoost, Random Forest, Logistic Regression, GBM, SHAP (XAI)
[31]	Riveros Perez E. (2025)	United States	29,509	Retrospective, population-level source	XGBoost, Random Forest, Logistic Regression, Decision Tree, SVM, CatBoost, GBM, SHAP (XAI)
[40]	Barman B. (2025)	India	1,087,006	Cross-sectional, population-level source	XGBoost, Random Forest, Logistic Regression, Decision Tree, SVM, CatBoost, GBM, KNN, SHAP (XAI)
[41]	Wang L. (2025)	China	1117	Retrospective, single-centre	Random Forest, Logistic Regression, Decision Tree, SVM, GBM, AdaBoost, Naive Bayes, KNN, SHAP (XAI)
[42]	Adams A.S. (2025)	United States	48,209	Retrospective, multicentre	Random Forest, Logistic Regression, Super Learner
[43]	Rahman M.H. (2026)	United States	4589	Prospective, population-level source	XGBoost, SVM, LightGBM, GBM, Cox/Survival ML
[30]	Carletti M. (2025)	United States	347 (of 1137)	Prospective, population-level source	XGBoost, Decision Tree, GBM, SHAP (XAI)
[44]	Nicolucci A. (2025)	Italy	138,558 eligible	Prospective, multicentre	XGBoost, GBM
[45]	Lin Z. (2025)	China	140	Retrospective, single-centre	Cox/Survival ML
[36]	Aminnejad N. (2025)	Canada	15,368	Retrospective, population-level source	XGBoost, GBM, Neural Network (ANN), CNN, Deep Learning (DL)
[35]	Lee K.A. (2025)	South Korea	5120	Retrospective, multicentre	XGBoost, Random Forest, SVM, Neural Network (ANN), SHAP (XAI)
[46]	Deng Z. (2026)	China	1049	Retrospective, provenance not stated	Logistic Regression
[47]	Gu J. (2026)	China	28,443	Retrospective, single-centre	Logistic Regression, Cox/Survival ML
[48]	Navarro-González J.F. (2026)	Spain	316,597	Retrospective, multicentre	XGBoost, Random Forest, Logistic Regression, Decision Tree, GBM
[49]	Chen M. (2025)	China	1912	Retrospective, multicentre	Logistic Regression
[50]	Liu X. (2025)	China	3755	Retrospective, single-centre	XGBoost, Random Forest, Logistic Regression, Decision Tree, SVM, SHAP (XAI)
[51]	Song M. (2026)	China	1124	Retrospective, single-centre	XGBoost, Random Forest, Logistic Regression, Decision Tree, SVM, CatBoost, LightGBM, GBM, Neural Network (ANN), SHAP (XAI)
[52]	Ma R. (2026)	China	296	Retrospective, single-centre	Logistic Regression, Deep Learning (DL)
[53]	Li Y. (2025)	China	1984	Retrospective, multicentre	XGBoost, SVM, LightGBM, SHAP (XAI)
[54]	Cobuz C. (2026)	Romania	150	Cross-sectional, single-centre	Neural Network (ANN)
[55]	Liu Y. (2026)	China	367	Retrospective, single-centre	Random Forest, Logistic Regression, SHAP (XAI)
[56]	Salim S.S. (2026)	Turkey	101,766	Retrospective, population-level source	XGBoost, Random Forest, Logistic Regression, LightGBM, GBM, SHAP (XAI)
[57]	Hu Y. (2025)	China	833	Retrospective, single-centre	XGBoost, Random Forest, Logistic Regression, Decision Tree, SVM, LightGBM, GBM, SHAP (XAI)
[58]	Wang L. (2026)	China	870	Retrospective, single-centre	XGBoost, Random Forest, Logistic Regression, SVM, LightGBM, GBM, AdaBoost, Neural Network (ANN), CNN, SHAP (XAI)
[59]	Anteneh A. (2025)	Ethiopia	9030	Cross-sectional, population-level source	Random Forest, Logistic Regression, Decision Tree, SVM, GBM, Neural Network (ANN)
[60]	Liu Y. (2025)	China	564	Hybrid (retrospective and prospective enrolment), multicentre	XGBoost, Random Forest, Logistic Regression, SVM, GBM, KNN, SHAP (XAI)
[61]	Navarro-González J.F. (2026)	Spain	353,371	Retrospective, multicentre	XGBoost, Random Forest, Logistic Regression, Decision Tree, GBM
[62]	Htoo P.T. (2025)	United States	14,776/10,679	Retrospective, population-level source	XGBoost, Decision Tree, GBM
[37]	Zhang Y. (2025)	China	6578	Retrospective, multicentre	XGBoost, Random Forest, Logistic Regression, Decision Tree, SVM, CatBoost, LightGBM, GBM, SHAP (XAI)
[63]	Luo J. (2025)	United States	14,436	Retrospective, multicentre	XGBoost, Decision Tree, GBM
[64]	Taschereau A. (2026)	Canada	1024/2670	Prospective, population-level source	Random Forest
[65]	Tan J. (2025)	China	Not stated	Retrospective, multicentre	Logistic Regression, Cox/Survival ML
[66]	Wongrith P. (2025)	Thailand	N/a (review)	N/a (scoping review)	None; scoping review of 26 conventional risk-screening tools (THAIRISK, CANRISK, FINDRISC, ADA-Risk)
[33]	Bernstorff M. (2025)	Denmark	74,880	Retrospective, population-level source	XGBoost, Logistic Regression, GBM
[38]	Koga D. (2025)	Japan	16,766	Retrospective, single-centre	XGBoost, Random Forest, Logistic Regression, LightGBM, GBM, Neural Network (ANN), SHAP (XAI)
[67]	Kashani M. (2026)	Iran	1284	Retrospective, multicentre	XGBoost, SVM, GBM, Neural Network (ANN), SHAP (XAI)
[29]	Afolabi S. (2025)	United States	100,000	Retrospective, population-level source	Random Forest, Logistic Regression, Decision Tree, KNN, SHAP (XAI)
[68]	Wang S. (2025)	Hong Kong	1810	Cross-sectional, population-level source	None; conventional risk scores only (FINDRISC, AUSDRISK, Chinese Diabetes Risk Score, ADA, USPSTF)
[69]	Zhou Z. (2026)	China	3836	Retrospective, single-centre	XGBoost, Random Forest, Logistic Regression, SVM, LightGBM, GBM, Naive Bayes, KNN, SHAP (XAI)
[70]	Zu Y.-Z. (2025)	China	426	Retrospective, single-centre	Logistic Regression
[71]	Li B. (2025)	China	619	Retrospective, single-centre	Cox/Survival ML
[32]	Yang H. (2026)	China	900	Retrospective, multicentre	XGBoost, Random Forest, Decision Tree, SVM, GBM, Naive Bayes, KNN, SHAP (XAI)
[72]	Zhong C.C. (2025)	China	2047	Retrospective, population-level source	KNN, Cox/Survival ML, SHAP (XAI)
[73]	Remedios L.W. (2025)	United States	1728	Retrospective, single-centre	Random Forest, Logistic Regression, Neural Network (ANN), SHAP (XAI)
[74]	Liu L. (2026)	China	10,163	Prospective, population-level source	Cox/Survival ML
[75]	Handley D. (2025)	United Kingdom	12,435	Prospective, population-level source	Logistic Regression, Cox/Survival ML
[76]	Ooko S.O. (2025)	Kenya	768	Retrospective, single-centre	Logistic Regression, Decision Tree, SVM, KNN, Neural Network (ANN)

**Table A2:** Data source, comparator, validation strategy and headline metric of the 47 included studies. Every value was verified against the full text of the study; the metric column states the data partition it comes from, because several studies report a training or derivation figure as their headline result. Target population is not tabulated here: it was not verified for all 47 with the same procedure, and is described per clinical domain in Section 4.3.

Ref.	Data Source	Comparator	Validation Strategy	Headline Metric
[34]	Single-centre (tertiary hospital, Shanghai)	Narrative/published values	Internal only	AUC 0.951 (XGBoost, test; 0.973 training)
[31]	NHANES 2007–2018	Narrative/published values	Internal only	AUC 0.8168 (XGBoost)
[40]	NFHS-5 national survey (India)	None (models compared with each other)	Internal only	AUC 0.6786 (RF, grid search)
[41]	Single-centre (PLA General Hospital)	Narrative/published values	Internal only	AUC 0.9731 (LR, highest); RF best overall: accuracy 96.7%, AUC 0.96
[42]	Two Kaiser Permanente regions	None (models compared with each other)	Internal only	AUC 0.845 (Super Learner; 95% CI 0.826–0.863)
[43]	All of Us Research Program	Conventional reference, same cohort	Internal only	C-index 0.867 (clinical Cox + steps + PRS); best single model 0.859
[30]	PROGRESS cohort (external: HPP)	Narrative/published values	External, independent cohort	AUC 0.96 (multimodal XGBoost, internal)/0.90 external
[44]	38 Italian diabetes clinics	Conventional reference, same cohort	External, independent cohort	AUC > 0.80 across complications; 0.97 nephropathy; accuracy > 70%
[45]	Single-centre (Fujian Medical University)	None (models compared with each other)	Internal only	C-index 0.831 (nomogram); time-dependent AUC 0.898/0.889/0.876 at 1, 2, 3 years
[36]	Diabetes Action Canada national repository	Conventional reference, same cohort	Internal only	AUC 0.88 (full model); 0.86 with significant variables only
[35]	Six hospitals (Korea)	None (models compared with each other)	External, independent cohort	AUC 0.8099 (XGBoost, development)/0.8113 external
[46]	Not stated (community health checks, Shenzhen)	None (models compared with each other)	Internal only	AUC 0.725 (nomogram, TRAINING)/0.698 validation
[47]	Single-centre (Banan Hospital, Chongqing)	None (models compared with each other)	Internal only	AUC 0.845 (training)/0.853 (validation)
[48]	Eight Spanish hospitals	None (models compared with each other)	External, independent cohort	AUC-ROC 0.72 (logistic regression, 27 predictors)
[49]	Two hospitals (China)	None (models compared with each other)	External, independent cohort	AUC 0.828 (derivation)/0.808 (internal)/0.788 (external)
[50]	Single-centre (First Hospital of Jilin University)	Narrative/published values	Internal only	AUC 0.85 (SVM, test)/0.82 (training); LR 0.824 in cross-validation
[51]	Single-centre (ophthalmology department)	None (models compared with each other)	Internal only	AUC 0.879 (SVM, validation); XGB 0.908 in derivation
[52]	Single-centre (People’s Hospital of Baoan, Shenzhen)	None (models compared with each other)	Internal only	None reported (odds ratios only)
[53]	Multicentre	None (models compared with each other)	Internal only	AUC-ROC 0.894 (LR, the model selected; 95% CI 0.814–0.964)
[54]	Single-centre (diabetes and metabolic diseases department)	None (models compared with each other)	Internal only	AUC 0.97 (CAN score) and 0.98 (nephropathy score), neural networks
[55]	Single-centre (Cangzhou Hospital)	Narrative/published values	Internal only	AUC 0.786 (CCR2 + IDO1 combined; 0.728 and 0.749 separately)
[56]	UCI Diabetes 130-US Hospitals dataset	Narrative/published values	Internal only	AUC 0.688 (calibrated stacking; 0.664 nested cross-validation)
[57]	Single-centre (neurology department)	None (models compared with each other)	Internal only	AUC 0.78 (Random Forest; F1 0.70)
[58]	Single-centre	Narrative/published values	Internal only	AUC 0.867 (stacking, validation; 95% CI 0.830–0.904)
[59]	Ethiopian Public Health Institute	None (models compared with each other)	Internal only	AUC 0.96 (GBM; accuracy 93%)
[60]	Three centres (Nanjing area)	None (models compared with each other)	External, independent cohort	AUC 0.806 (nomogram, training)/0.861 external
[61]	Eight Spanish hospitals	None (models compared with each other)	Internal only	AUC-ROC 0.73 (logistic regression, the model selected)
[62]	Medicare registry (external: Optum Clinformatics)	Narrative/published values	External, independent cohort	c-statistic 0.75 (LASSO, with baseline CVD)/0.73 (without)
[37]	Two Chinese medical centres	Narrative/published values	External, independent cohort	AUC 0.819 (CatBoost, test)/0.807 external
[63]	MedStar Health System (10 hospitals)	None (models compared with each other)	Internal only	AUC 0.8159 (test cohort; 95% CI 80.40–82.72%)
[64]	Gen3G cohort (external: Project Viva)	None (models compared with each other)	External, independent cohort	ROC-AUC 0.696 (Gen3G, cross-validated)/0.682 (Project Viva)
[65]	Three hospitals (Wuxi)	None (models compared with each other)	Internal only	AUC 0.705 (nomogram, TRAINING)/0.666 (test); three models ~0.64
[66]	n/a (scoping review of 26 tools)	Conventional reference, same cohort	Internal only	AU-ROC, sensitivity and specificity of the 26 tools reviewed; no model of its own
[33]	Central Denmark Region psychiatric services	Conventional reference, same cohort	Internal only	AUROC 0.84 (XGBoost, test)
[38]	Single clinic (Tokyo)	None (models compared with each other)	External, independent cohort	AUROC 0.851 (internal test)/0.785 external; 0.844 single-lead
[67]	Two hospitals (Sirjan)	None (models compared with each other)	Internal only	Accuracy 94% (hybrid DNN + XGBoost); precision 93%, recall 95%
[29]	Public CDC dataset	None (models compared with each other)	Internal only	Accuracy 96.09% (KNN); sensitivity 98.54%, specificity 93.63%; no AUC
[68]	Community screening programme (Hong Kong)	Conventional reference, same cohort	Internal only	Sensitivity 96.6%, specificity 19.2% (NICE criteria); strategies range 19.5–100%
[69]	Single-centre (Nanjing Drum Tower Hospital)	None (models compared with each other)	Internal only	AUROC 0.873 (XGBoost; AUPRC 0.904)
[70]	Single-centre (Chongqing Hospital of TCM)	None (models compared with each other)	Internal only	AUC 0.826 (nomogram); bootstrap-corrected C-index 0.795
[71]	Single-centre (Air Force Medical Center, Beijing)	Conventional reference, same cohort	Internal only	AUC 0.806 (marker + base model at 365 days); 0.656–0.661 markers alone
[32]	Two tertiary hospitals	None (models compared with each other)	External, independent cohort	AUC 0.89 (Random Forest, internal test)/0.83 external; 0.914 training
[72]	Territory-wide registry (Hong Kong)	None (models compared with each other)	Internal only	C-index 0.90 (random survival forest); time-dependent AUC 0.823
[73]	Single-centre (Vanderbilt University Medical Center)	None (models compared with each other)	Internal only	Mean AUC 0.72–0.74 (Random Forest, by BMI stratum)
[74]	METSIM cohort (Finland)	Conventional reference, same cohort	Internal only	AUC 0.739 (DDRS + metabolites); 0.727 risk factors + metabolites
[75]	UK Biobank	None (models compared with each other)	Internal only	None reported (hazard and odds ratios only)
[76]	Single clinic (Africa)	None (models compared with each other)	Internal only	ROC-AUC 0.91 (optimised neural network); accuracy 89.0%

**Table A3:** PROBAST + AI (model-development part) assessment of the eight anchor studies: concern regarding quality by domain and overall, and applicability to the review question. D1, participants and data sources; D2, predictors; D3, outcome; D4, analysis; P, participants; Pr, predictors; O, outcome. The assessment was performed once, from a structured extraction of the relevant passages of each full text, and not in duplicate (Section 2.7); the answers to the 16 signalling questions for each study are provided as Supplementary Table S1.

Ref.	Study	Quality D1/D2/D3/D4	Overall Quality	Applicability P/Pr/O	Overall Applicability	Main Concerns
[34]	Peng et al. (2026)	High/Low/High/Low	High	Low/Low/Low	Low	Single-centre convenience sample; prevalent DPN modelled as risk; blinding not reported
[31]	Riveros Perez and Avella-Molano (2025)	High/Low/High/High	High	Low/Low/High	High	Cross-sectional; self-reported outcome incl. “borderline”; complete-case analysis
[40]	Barman et al. (2025)	High/Low/High/High	High	Low/Low/Low	Low	Cross-sectional; SMOTE without recalibration, applied before the split; complete-case
[41]	Wang et al. (2025)	High/Low/Low/High	High	Low/Low/Low	Low	Single-centre, men only, 1998–2005 data; listwise deletion; model chosen on the reporting set
[42]	Adams et al. (2025)	Low/Low/Low/Low	Low	Low/Low/Low	Low	None in any domain; internal validation only
[43]	Rahman et al. (2025)	High/Low/Low/High	High	Low/Low/Low	Low	Selection toward wearable users; complete-case eligibility; 265 events for a 12-model suite
[30]	Carletti et al. (2025)	High/Low/High/High	High	Low/Low/High	High	Self-reported status as label; cross-sectional target; 268 participants for a multimodal model
[44]	Nicolucci et al. (2025)	Unclear/Unclear/Unclear/Unclear	Unclear	Low/Low/Low	Low	Implementation study: development reported in its own reference only
